# Achiral Nanoparticle-Enhanced Chiral Twist and Thermal Stability of Blue Phase Liquid Crystals

**DOI:** 10.1021/acsnano.2c07321

**Published:** 2022-12-08

**Authors:** Kamil Orzechowski, Martyna Tupikowska, Olga Strzeżysz, Ting-Mao Feng, Wei-Yuan Chen, Liang-Ying Wu, Chun-Ta Wang, Eva Otón, Michał M. Wójcik, Maciej Bagiński, Piotr Lesiak, Wiktor Lewandowski, Tomasz R. Woliński

**Affiliations:** † Faculty of Physics, Warsaw University of Technology, Koszykowa 75, 00-662Warsaw, Poland; ‡ Faculty of Chemistry, University of Warsaw, Pasteura 1, 02-093Warsaw, Poland; § Institute of Chemistry, Military University of Technology, Kaliskiego 2, 00-908Warsaw, Poland; # Department of Photonics, National Sun Yat-sen University, No. 70 Lien-hai Road, Kaohsiung80424, Taiwan; ◊ Institute of Applied Physics, Military University of Technology, Kaliskiego 2, 00-908Warsaw, Poland

**Keywords:** liquid crystals, blue phases, gold nanoparticles, ligands, Bragg reflection, thermal stabilization

## Abstract

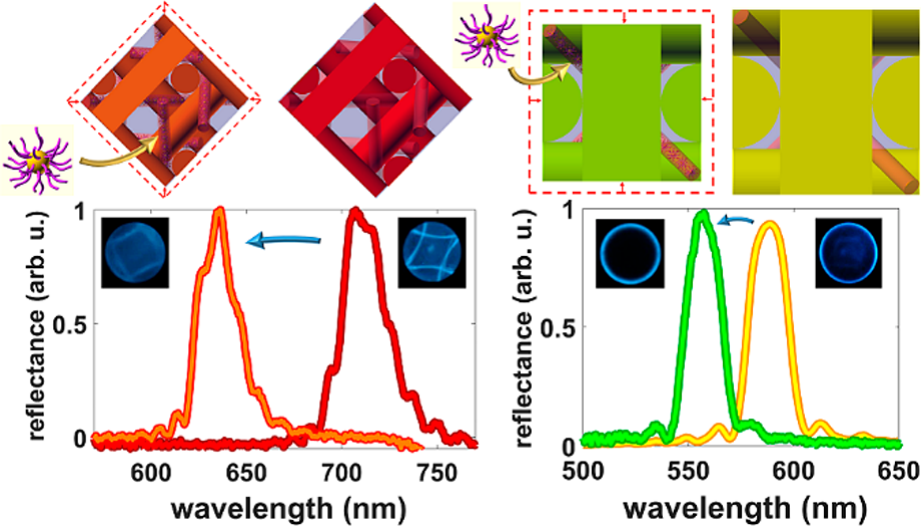

Blue phase liquid crystals (BPLCs) are chiral mesophases with 3D order, which makes them a promising template for doping nanoparticles (NPs), yielding tunable nanomaterials attractive for microlasers and numerous microsensor applications. However, doping NPs to BPLCs causes BP lattice extension, which translates to elongation of operating wavelengths of light reflection. Here, it is demonstrated that small (2.4 nm diameter) achiral gold (Au) NPs decorated with designed LC-like ligands can enhance the chiral twist of BPLCs (i.e., reduce cell size of the single BP unit up to ∼14% and ∼7% for BPI and BPII, respectively), translating to a blue-shift of Bragg reflection. Doping NPs also significantly increases the thermal stability of BPs from 5.5 °C (for undoped BPLC) up to 22.8 °C (for doped BPLC). In line with our expectations, both effects are saturated, and their magnitude depends on the concentration of investigated nanodopants as well the BP phase type. Our research highlights the critical role of functionalization of Au NPs on the phase sequence of BPLCs. We show that inappropriate selection of surface ligands can destabilize BPs. Our BPLC and Au NPs are photochemically stable and exhibit great miscibility, preventing NP aggregation in the BPLC matrix over the long term. We believe that our findings will improve the fabrication of advanced nanomaterials into 3D periodic soft photonic structures.

Over the last two decades, blue phase liquid crystals (BPLCs) have attracted tremendous interest due to prospective, beyond-display, applications in advanced photonic technologies, enabling fabrication of (i) Bragg–Berry holograms with omnidirectional circular-polarization selectivity,^1^ (ii) flexible and tunable mirrorless lasers,^2,3^ (iii) erasable^4^ and bioinspired photonic color coatings,^5^ and (iv) long-period^6^ and tunable^7^ photonic fibers for ultrafast communication. These applications were unlocked due to the fascinating supramolecular, hierarchical structure of BPLCs, which comprise chiral, double-twist cylinders (DTCs), arranged into body-centered cubic (BPI) and simple cubic (BPII) lattices with a few hundred nanometers’ unit cell size.^8,9^ Such structures translate to strong interactions with visible light, resulting in vivid reflected coloring due to a 3D photonic bandgap^10,11^ and an optical rotatory power due to their optical activity.^12^ However, properties such as macroscopic optical isotropy and polarization insensitivity are revealed in BPLC materials for wavelengths outside the resonance band.^13,14^ Such hierarchical structure translating to both chiral and photonic properties is usually only found in complex natural materials, such as the cuticle of *Chrysina gloriosa*(15) or the plant tissue of *Pollia* fruit,^16^ making BPLCs highly interesting biomimetic materials. The key feature of BPLCs in this context is the direct interplay between the chiral properties determined by the DTC and photonic properties determined by the BP cubic symmetry. Namely, the unit cell size is equal to either a full or a half pitch of the DTC, for BPI and BPII, respectively. For applications, increasing the thermal stability and achieving controlled photonic properties are highly desirable. These features of BPLC materials make them particularly promising for photonic technologies in which BPLCs act as a soft, organic host for organic or nanoscale materials. However, achieving BPLC composites with broadened thermal stability without deteriorating photonic properties is challenging.

To answer the above challenge, it is worth noting that neighboring DTCs, forming a 3D cubic lattice, cannot merge without maintaining the twisted LC director, causing the formation of a network of disclinations. These places of molecular misalignment are the most energetically disfavored regions contributing to the existence of BPs in a relatively narrow range of temperatures (∼0.1–5.0 °C). Nevertheless, previous experimental studies proved that due to the isotropic core of the defects, they can be effectively used as a 3D periodic host for a variety of polymers,^17−20^ rod-like molecules,^21^ and nanomaterials, such as dielectric,^22−28^ magnetic,^29,30^ and semiconductor^31−33^ nanoparticles (NPs), carbon nanotubes,^33^ or graphene oxide.^34−36^ Unfortunately, these additives have been primarily investigated as agents stabilizing BPs,^17−23,25,27−36^ with limited focus on tuning their influence on photonic properties of BPLC materials^24,28^ (e.g., selective reflections) that is of particular interest for future photonic applications of BPLCs. If tested, usually additives decrease the twist of the DTC, increasing helical pitch.^28^ Whether increasing the DTC twist via placing nanoinclusions in disclination lines to broaden the available wavelength range is possible remains to be determined.

Another challenge in designing photonic devices based on BPLCs is achieving selective reflection over large areas. One approach relies on the pinning effect where either boundary conditions of the LC-cell substrate or the tiny cell gap size (<1 μm) affects BP crystal ordering and/or unit cell size,^37^ controlling over a wavelength of selective reflection. Whereas great thermal stabilization can be achieved through polymer (Δ*T* ∼60 °C)^19^ or rod-like molecule doping (Δ*T* ∼132 °C),^21^ these systems do not offer the advantage of photonic property modification.^19−21^ Recently, a 3D cubic lattice deformation under electrostriction was investigated by applying an external electric field, causing both BP lattice structure and field-induced phase transitions.^38^ Unfortunately, the above methods cannot increase stability and simultaneously adjust the photonic bandgap of BPLCs, while NP doping could potentially resolve these issues.

A decade ago, Ravnik et al.^39^ theorized that infiltrating BPLCs with NPs (NP/BPLC composite) may be an efficient tool for achieving expansion or contraction of the cubic BPs’ unit cell. The action of NPs was shown to be dependent on the particle size and surface anchoring properties of NPs determining the particle–LC tension and driven by changes in the energetic cost of LC disclinations, as explained by the theory of elasticity. It was shown that the relative size of the BP unit cell can be easily and significantly expanded by doping with larger particles (>100 nm diameter). However, a contraction of the relative unit cell can be only less than 2% by doping with comparatively medium-sized (∼20–100 nm diameter) colloidal NPs, assuming even relatively low surface anchoring energies of NPs (*W* ∼10^–6^ J/m^2^). It is worth noting that the calculations were done for fully wetted surfaces of NPs where particle–liquid crystal interfacial tension can be ignored. In this work, we propose very small NPs with properly designed coating and size to overcome the limitations related to the contraction of the cubic BPs’ unit cell.

In this paper, we demonstrate that achiral gold (Au) NPs can simultaneously enhance the twist of chiral cylinders and the thermal stability of cubic BPs, without deteriorating the possibility of large area selective reflection. A set of NPs with different types of ligands, ensuring proper anchoring properties of NPs, were designed and tested as nanoinclusions to BPLCs. When doping nanomaterials to BPLC or other organic hosts, phase segregation and stability are often crucial issues.^40^ These issues are also addressed by the NP coating we propose. We show that NPs with an LC-like organic shell may reduce the size of the BP unit cell, translating to tunable Bragg reflections, while significantly increasing the thermal stability of blue phases. Both essential features of the self-assembled soft photonic structures alter with the concentration of NPs, expanding the range of possibilities in a design of spectrally adjustable photonic devices with a broad tuning in the visible range.

## Results and Discussion

To prepare the NP/BPLC composite we decided to use induced BPLCs formed by doping nematics with a strongly twisting chiral dopant. As the nematic LC base, we used photochemically stable fluorinated oligophenyls with fluorinated cyclohexyl- and bicyclohexylbiphenyls (85.8 wt %).^41^ This choice was dictated by the low-birefringence and temperature-dependent characteristic typical for nematics, not complicated by additional nematic–nematic phase transition (Figure S1). The BP was induced by the addition of two chiral dopants: biphenyl-4,4-dicarboxylic acid bis(1-methyl heptyl) ester (7.0 wt %) and [1,1;4,1] terphenyl-4,4-dicarboxylic acid bis(1-methyl heptyl) ester (7.2 wt %).^42^ The helical pitch of the investigated BPLC is 350 nm, measured at 25 °C in the chiral nematic (N*) phase with the use of the Grandjean–Cano method (Figure S2). Chemical structural formulas and other measured macroscopic electro-optic parameters of individual constituents of the investigated BPLC can be found elsewhere.^43^

As nanoinclusions we decided to use spherical Au NPs (2.4 ± 0.3 nm diameter) due to the high chemical and thermal stability as well as the well-known chemistry of Au nanocrystals. Nanoparticles covered with a dodecanethiol coating (Au@DDT) were prepared following a modified Brust–Schiffrin method. Then, about half of the dodecanethiol ligands were partially replaced in a ligand exchange reaction, with one of liquid-crystal-like thiols—4-((12-((11-sulfanoundecanoyl)oxy)dodecyl)oxy)phenyl-4-(octadec-9-en-1-yloxy)benzoate (L1) or [(4′-{[4-(dioctylcarbamoyl)phenyl]methoxy}-[1,1′-biphenyl]-4-yl)oxy]undecyl 11-sulfanylundecanoate (L2)—yielding Au@L1 and Au@L2 nanomaterials, respectively. Notably, the surface docking of used ligands is strong, due to the strong nature of S–Au interactions.^44^ It is important to note that Au@L2 NPs have a larger alkyl portion that can spherically interact with the LC matrix more efficiently and amide bonds capable of producing strong dipole–dipole interactions. Details of ligand exchange reactions are given in the Materials and Methods section, and the characterization of the investigated NPs can be found in Figure S3.

It is worth appreciating the rod-like and aromatic structure of LC-like ligands introduced into the nanocrystal organic shells, which favors steric and π–π interactions with the LC molecules forming BP. Simultaneously, the binary type of ligand shell, comprising larger and smaller ligands, renders the organic shell flexible, allowing it to adapt to the host geometry. A direct proof of this capability is provided by drop-casting and heat annealing NPs without a BP matrix. X-ray diffraction studies attest that both Au@L1 and Au@L2 NPs form ordered assemblies with interparticle periodicities ∼7–8 nm and ∼3–4 nm in orthogonal directions, in analogy to our previous research on similar systems.^45,46^ The formation of these lamellar structures was driven by tactoidal deformation of the organic shell. The binary monolayer of ligands (alkyl + LC-like) has been already shown to favor the dispersibility of NPs within the LC matrix, e.g., in the nematic phase^47^ or helical nanofilament phase.^48^ In the latter case, the tendency of NPs to locate at the phase boundaries/defects of LC materials was evidenced, thought to originate from lowering the total energy of the system, probably by decreasing the molecular order at the helical nanofiber edges, caused by admixing of mesogenic molecules from the NPs’ grafting layer. Thus, we expected that the as-designed surface coating should enable placing Au NPs at, and flexible adaptation of, Au NPs to the disclination areas when doped to the BPLCs. In other words, we expected LC-like ligands to act as spatial stabilizers of such an arrangement that is forced by the anchoring conditions on the surface of nanocrystals.

**Figure 1:**
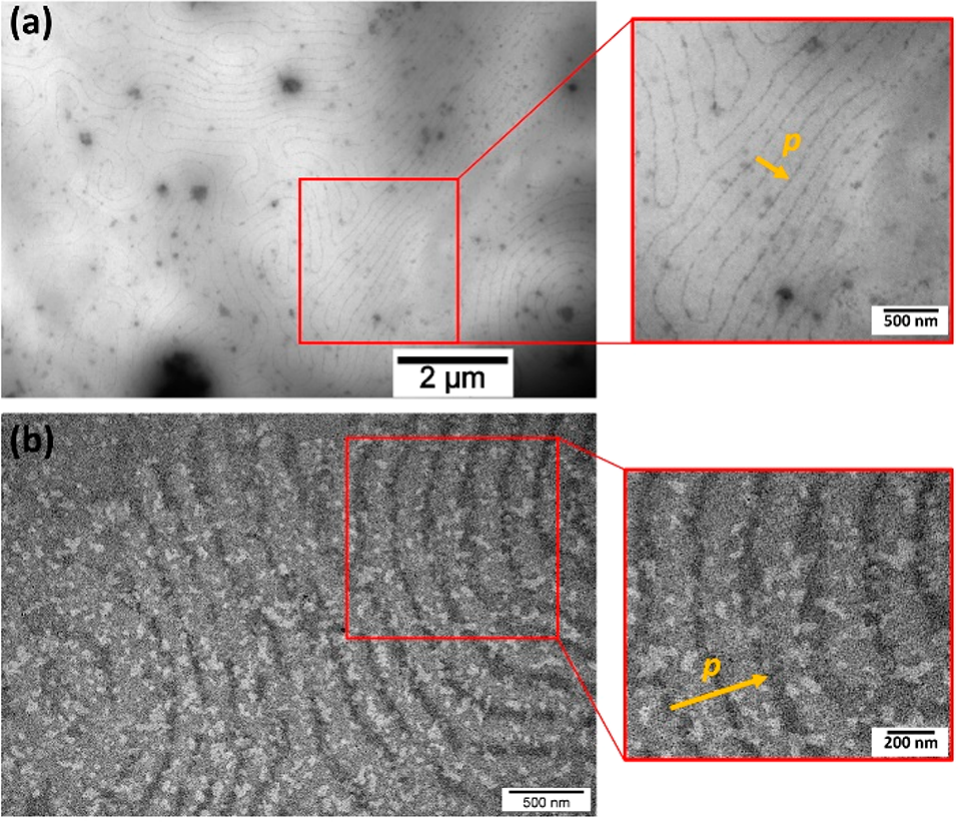
TEM images of BPLC (in N* phase) doped with Au@L1 of 0.5 wt % (a) and Au@L2 of 2.0 wt % (b). The helical pitch is shown on the enlarged area as a distance between two lengths of neighboring dark lines.

Mixtures of BPLC and NPs were prepared by mixing toluene dispersions, drop-casting, and heat annealing. We first probed materials using transmission electron microscopy (TEM) to confirm that NPs do not form large, phase-separated aggregates and verify if they adopt an anisotropic distribution, suggesting that NPs accumulate at LC defects. Since BP forms only at elevated temperatures, making it difficult to be probed using TEM, we relied on observing interactions between NPs and the N* phase of the investigated BPLC material (Figure 1) which forms at temperatures below BP. TEM images acquired at a low, 0.5 wt %, Au@L1 NP concentration revealed that NPs are spotted in areas concerned with organics, suggesting good compatibility between BPLCs and NPs. At 2.0 wt % doping Au@L2 NPs were well distributed, covering the area of the sample. Notably, the distribution of Au@L1 and Au@L2 NPs within these composites was not random; instead, particles formed periodically arranged 1D linear assemblies that resemble the cholesteric fingerprint texture (Figure 1a,b). Previous studies suggest that the contrast observed in LC films by TEM is caused by different factors, but, in general, the contribution of the LC director is that it shows a bright image when it is perpendicular to the film and dark when parallel. Thus, the helical axis is perpendicular to the lines, and the distance between neighboring dark lines would correspond to the half-pitch of our mixture. Importantly, the contrast is further enhanced by NPs’ preferential placement in the parts in which the orientation of LC molecules is parallel to the film plane (dark lines), which agrees well with the results for NP-doped cholesteric liquid crystals achieved by Mitov et al.^49^ The measured helical pitch of BPLC samples doped with 0.5 wt % of Au@L1 NP and 2.0 wt % of Au@L2 corresponds to 316 and 448 nm, respectively (Figure S4), which implies a significant influence of Au NPs on the chiral twist of the investigated BPLC material. Although TEM observations were performed for the N* phase, that is, at temperatures lower than those required for BP of the studied samples, acquired results strongly support the idea that Au@L1 and Au@L2 nanodopants should localize in LC disclinations of the blue phase.

We next used polarized optical microscopy (POM) in transmission with crossed polarizers to investigate the formation of BP in purely organic and composite samples comprising 0.5 wt % NPs. Mixtures were observed under POM on cooling from the isotropic phase (ISO). We decided to investigate the BPLC samples in the cooling process rather than heating due to the higher thermodynamic stability of BP phases.

**Figure 2:**
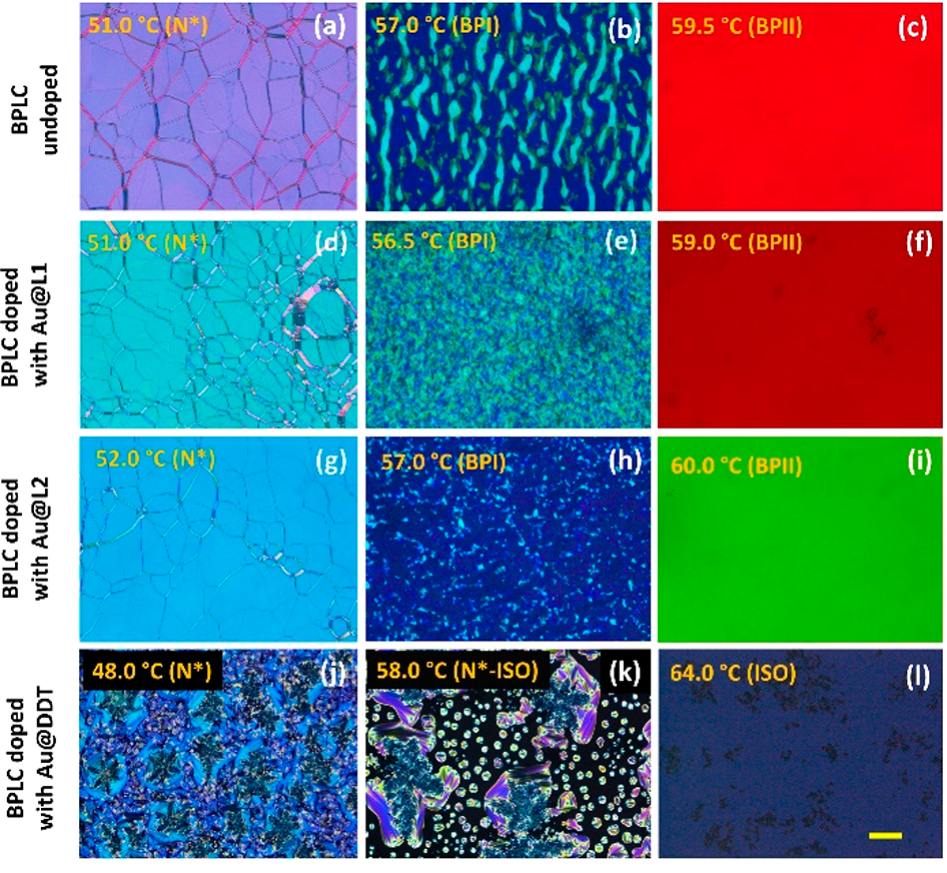
Polarized optical microscopy images of BPLC at different temperatures (LC phases): undoped (a–c) and doped with Au@L1 (d–f), Au@L2 (g–i), and Au@DDT (j–l). All samples have the same NP concentration of 0.5 wt % in the LC mixture. The yellow scale bar corresponds to 100 μm.

For the undoped sample, BPI exhibits selective reflection for blue/green light (Figure 2b), while BPII for red light (Figure 2c). Their thermal stability is in the range of 3.5 °C (54.5–58.0 °C) and 2.0 °C (58.0–60.0 °C), respectively. In contrast, their stability on heating is much lower and is in the range of 0.4 °C (57.6–58.0 °C) for BPI and 2.6 °C (58.0–60.6 °C) for BPII. By using Au@L1, Au@L2, and Au@DDT NPs we hoped to get preliminary information on the effect of the composition of the organic shell around NPs on the formation of composites.

When analyzing composites, we noted that doping Au@L1 NPs to BPLC did not affect the sequence of appearing phases of the LC mixture, allowing us to record selective reflections characteristic of BPI and BPII (Figure 2e,f) appearing to be in the same reflection range as the undoped BPLC sample. The texture of BPI in the doped sample is more polycrystalline; for example, smaller cyan domains are formed (∼350 μm^2^) than BP platelets in undoped BPLC (∼2450 μm^2^; compare Figure 2b,e). Minute particle aggregation occurred after four months’ storage of the doped composite, visible as small dark spots (Figure S5). For the studied composite the thermal stability of BPsI and -II is in the range of 5.0 °C (52.2–57.2 °C) and 2.0 °C (57.2–59.2 °C), respectively.

In the case of doping Au@L2 NPs to BPLC, the sequence of phases showed that the Bragg reflection in BPII was shifted toward shorter wavelengths (Figure 2i) compared to the pure BPLC (Figure 2c). We will discuss this effect in detail in a later part of the text. Thermal stability of doped-BPsI and -II is in the range of 4.5 °C (55.0–59.5 °C) and 2.2 °C (59.5–61.7 °C), respectively. It is worth highlighting that in the studied sample particle aggregation has not occurred for more than one year, a crucial feature in view of practical applications.

In contrast to NPs comprising LC-like ligands, Au@DDT NPs mixed in the BPLC matrix exhibit an immediate and strong tendency to phase separate, forming dark-appearing aggregates. POM images revealed that in this case BPI and BPII phases were not formed; instead, a direct isotropic to N* phase transition was observed with a broad region of coexisting phases (Figure 2j–l).

Overall, the above-discussed POM analysis confirmed that the proposed binary ligand shell design of Au NPs enabled the fabrication of nanoinclusions that are able to efficiently mix with the BPLC. Among the tested materials Au@L2 exhibited the lowest tendency to form aggregates; thus we decided to test this material in more detail. We prepared Au@L2/BPLC composites comprising 0.5, 2.0, and 5.0 wt % doping to a BPLC mixture and studied their optical properties by POM in reflection mode. It is worth mentioning that slight differences from selective reflections recorded under crossed polarizers in transmission might originate from the optical activity of the BPLC.

**Figure 3:**
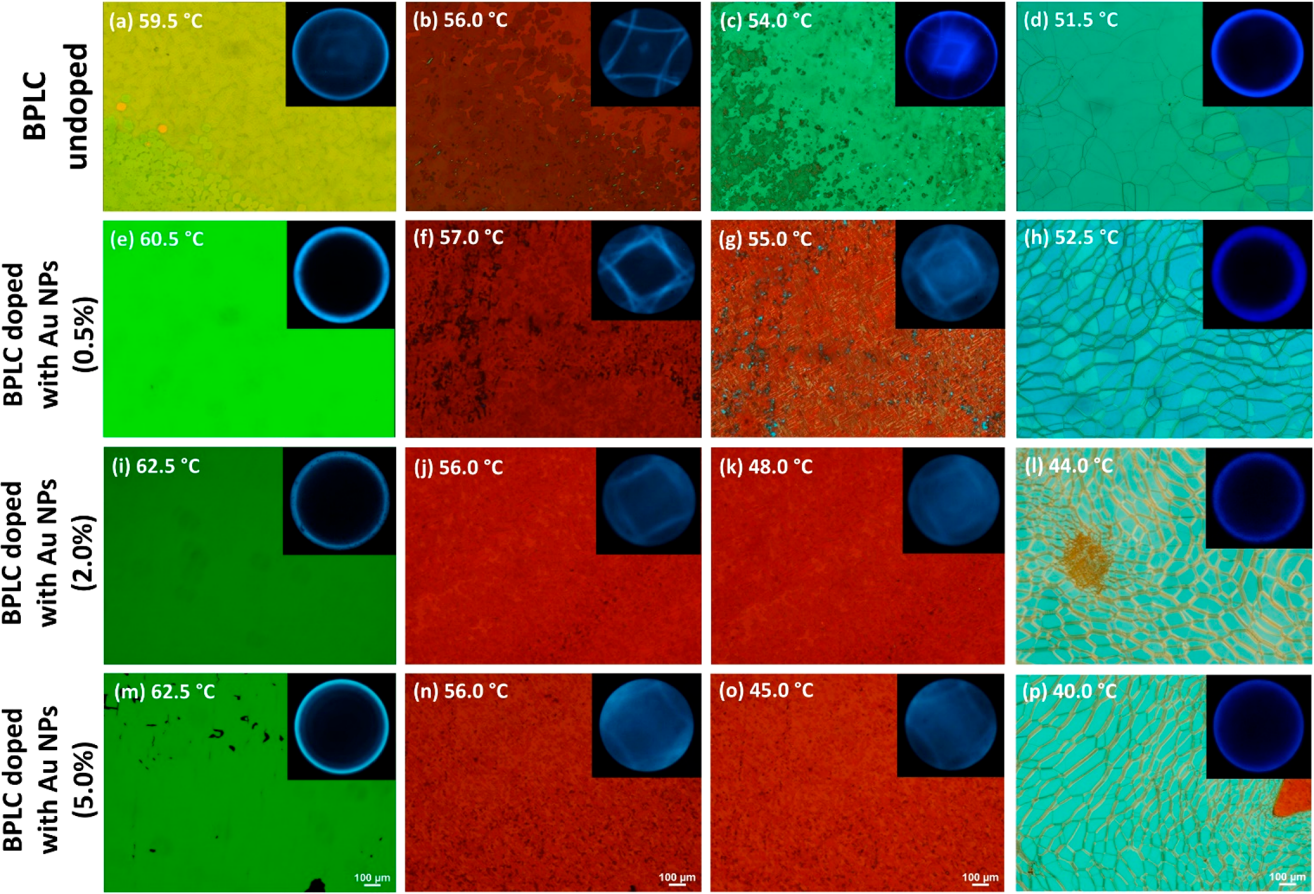
POM images of the undoped BPLC (a–d) and BPLC doped with Au@L2 NPs for different concentrations: 0.5 wt % (e–h), 2.0 wt % (i–l), 5.0 wt % (m–p). Optical textures were obtained in the reflection in a gradually cooling process from the isotropic phase. The insets correspond to the Kossel diagrams obtained for monochromatic light at 488 nm (fwhm = 10 nm), confirming the existence of either cubic BPs or N* phases at appropriate temperatures.

POM images recorded in reflection show quasi-monodomain textures of undoped BPsII and -I (Figure 3a,b) due to applied homogeneous alignment layers in the samples, which coincides with the results from other work.^50^ We can see dominant yellowish BP domains in BPII, while red and tiny cyan domains can be noticed in BPI. The existence of a continuous phase transition from BPI to N* occurs over a range of 3.0 °C (51.5–54.5 °C), shifting selective reflection toward shorter wavelengths during the cooling of the sample (Figure 3c). This behavior is characteristic of nematic LC mixtures with chiral dopants^51^ and might be interesting for lasing applications, providing two signals with only one of them sensitive to temperature. The POM images show a blue-shift of the selective reflections due to doping the BPLC with Au@L2 NPs, clearly noticeable for BPII (see, e.g., Figure 3a,e). Beyond affecting the phase transition temperature and selective reflection, these experiments show that doping NPs may affect domain orientation.

This effect was confirmed at NP-doped BPII, for which domains are aligned homogeneously with Bragg reflection for green light, whereas NP-doped BPI texture is quasi-monocrystalline, having dominant selective reflection for red light. Here, it can also be noticed the growth of grain boundaries in the texture as defects, indicating a mismatching in BP domain orientation over a large area of the sample (compare Figure 3b,f,j,n). However, this is not due to particle aggregation; the investigated NPs are well dispersed up to a concentration of 5.0 wt % in BPs. This is also confirmed by measurements of the spectra of white-light transmittance through the BPLC-doped samples in the isotropic phase (Figure S6).

Beyond POM images we also measured Kossel diagrams at temperatures characteristic of different phases, allowing us to investigate the nature of the phases, BP lattice orientation, the unit cell size of BP, and the relative degree of monocrystallinity.^52−54^ Here, in all cases BPsII and -I with cubic symmetry were found, having a space group of *O*^2^ (*P*4_2_32) and *O*^8^ (*I*4_1_32), respectively. The Kossel diagrams show that the pure BPLC sample exhibits (100) and (110) crystallographic orientations of BPII and BPI, respectively (Figure 3a,b). The measurements of the Kossel diagram also confirmed the existence of a continuous phase transition BPI-N*, showing simultaneously typical lines for BPI (110) and a single ring for the N* phase^55^ (Figure 3c). Moreover, it confirms the correlation between different experiments. Doping BPLC with Au@L2 NPs leads to a slight loss of monocrystallinity of the sample in BPI (compare Figure 3b,f,j,n). The Kossel pattern appears to be blurry or dim (not sharp, thin lines) possessing several lines intersecting each other for higher concentrations of nanodopants.

This is because the observed Kossel lines are an average of the thickness of the whole sample, and slight variations of BP crystal orientation, mainly occurring in the bulk, might not be visible in the POM images. Still, they would contribute to the loss of sharpness of Kossel lines. Overall, we can say that there are BP crystals with slightly different lattice orientations; however, the majority of BPI corresponds to a crystal plane with a Miller index (110) having variable azimuthal angles. In the case of BPII, a change of the Kossel pattern upon higher NP concentration is hard to note, owing to the characteristic donut-shape Kossel pattern of BPII (100).

Therefore, we decided to analyze the case of BPI considering the influence of Au@L2 NPs on the BP lattice structure by simulations of the Kossel diagram. Due to the above-mentioned issues and some difficulties in fitting exact lines to those from the experiment (e.g., Figure 3f,j,n), they are presented in a later part of the text after calculating the lattice constants of all BPLC samples based on the measurements of selective light reflections.

Overall, the detailed POM/Kossel diagram measurements confirmed that BPLCs retain their photonic properties after doping with NPs and that NPs have a substantial effect on the BP concerning the crystal plane orientation and unit cell size, with the latter clearly dependent on NP concentration. To unequivocally confirm these results, we decided to measure the Bragg reflection from the same set of samples. The measurements of Bragg wavelengths (λ_B_) allow one to determine the exact temperature of phase transitions and also the cubic lattice unit cell in an indirect way using the following eq 1:
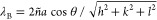
1in which *ñ* is the average BPLC refractive index, *a* is the cubic lattice constant, (*h k l*) are Miller indices of the crystal lattice, and θ is an incident angle of light.

**Figure 4:**
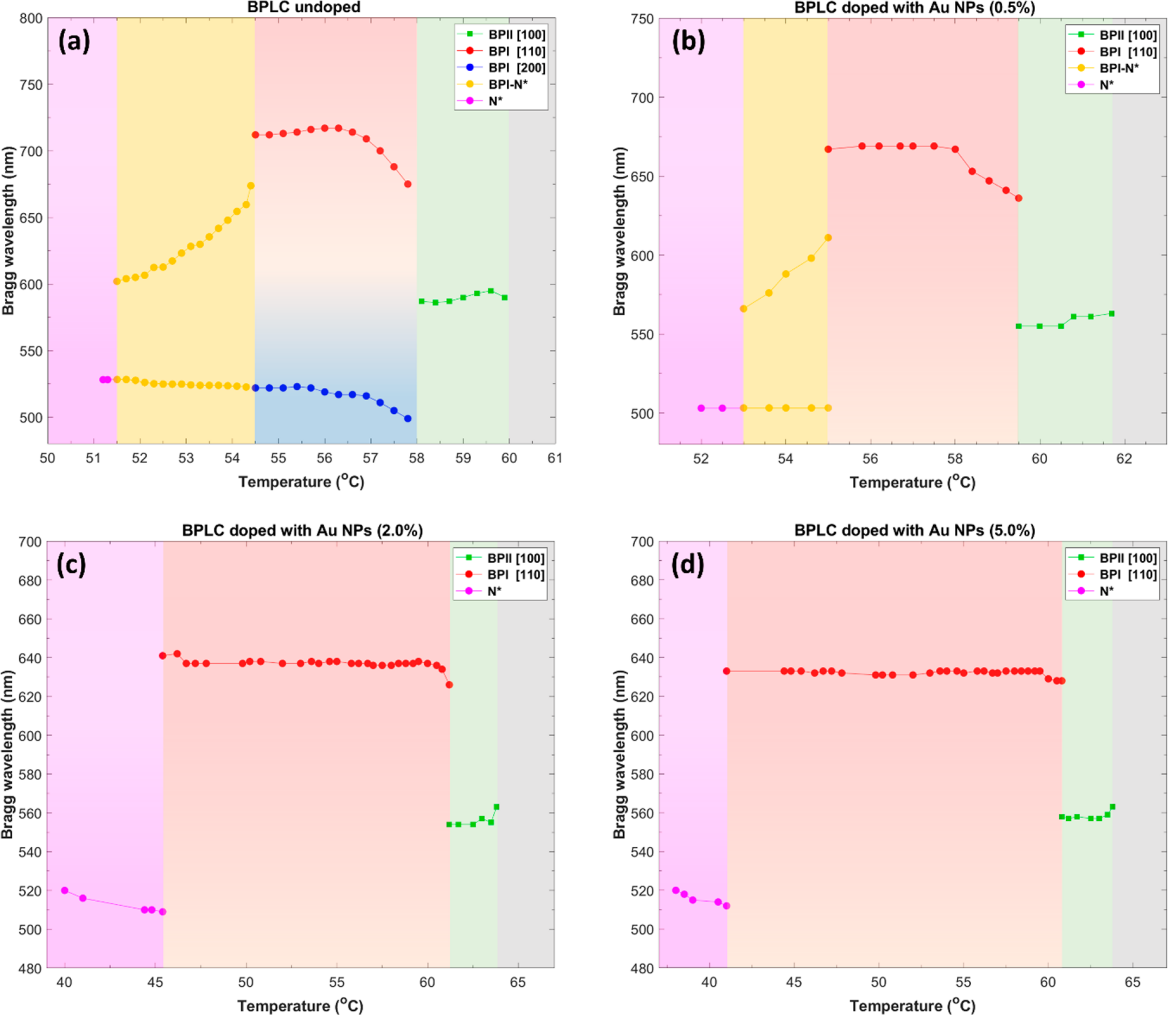
Temperature-dependent Bragg wavelengths for undoped BPLC (a) and BPLC doped with Au@L2 NPs for different concentrations: 0.5 wt % (b), 2.0 wt % (c), and 5.0 wt % (d) in cooling the investigated samples.

In the case of a purely organic BPLC, the Bragg wavelength of BPII (100) is around 595 nm, while two signals of (110) and (200) for BPI were measured in reflection at around 717 and 517 nm, respectively (Figure 4a).

The results also show an interesting jump in selective reflection near the BPI–N* phase transition for BPI (110) toward shorter wavelengths upon cooling the sample from BPI to N*, which corresponds to 38 nm (from 712 to 674 nm) (Figure 4a).

The measurements show that doping the BPLC with the investigated Au@L2 NPs leads to a blue-shift of the Bragg wavelength (Δλ_B_), and its value is greater upon higher NP concentration for both BPI and BPII phases. Only the reflection signal for BPI (200) has not been detected for NP-doped BPLC samples due to the orientation of BP domains by NP doping.

It seems that Δλ_B_ is significant even for an NP concentration of 0.5 wt % corresponding to 48 and 34 nm for BPI and BPII, respectively, giving a relative change of ∼6.7% for BPI and ∼5.7% for BPII (Figure 4b). Moreover, Au NPs of 0.5 wt % doped to BPLC lead to a blue-shift of Bragg wavelength for BPI (110) near the BPI–N* phase transition, corresponding to 56 nm (from 667 to 611 nm) (Figure 4b), simultaneously reducing the temperature range of BPI–N* up to 2.0 °C (53.0–55.0 °C), whereas for higher NP concentration the coexistence of BPI and N* phases does not occur. For an NP concentration of 2.0 wt %, Δλ_B_ increased distinctly for BPI to 80 nm and only slightly for BPII, to 38 nm, which corresponds to 11.2% and 6.4% of the relative Bragg wavelength change for BPI and BPII, respectively (Figure 4c). At 5.0 wt % of Au@L2 -doped BPLC, a slight blue-shift of λ_B_ only for BPI was noticed up to 84 nm (Figure 4d). It is worth noting that the most significant Bragg wavelength shift for BPII occurs for the lowest Au NP concentration tested, while for BPI, at 2.0 wt % of Au NPs. For higher Au NP concentrations, the relative Bragg wavelength shifting equaled 4 nm for both BPs, representing less than 1% of Δλ_B_ and finally reaching the saturation threshold.

This effect will be discussed in detail in a later part of the text. The values of Bragg wavelength shifting in BPs caused by doping of Au NPs are presented in Table S1, and the spectra of selective reflection measurements at the selected temperatures (LC phases) for all BPLC samples can be found in Figure S7.

We also noticed that the impact of the investigated Au NPs on the Δλ_B_ (see Figure 4) and the helical pitch in N* (Figure S8) is very distinct from those in BPs, regarding the saturation threshold and blue/red shift (Supplementary Note 1).

Before we calculated the cubic lattice constant in an indirect way based on eq 1, the influence of Au NPs on the average refractive index change of BPLC was checked by analyzing the characteristic signal with the amplitude modulated in the spectra of white light reflected from the BPLC samples and by using the following eq 2:
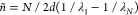
2in which *N* means the number of maxima/minima taken into calculation, *d* is the cell gap, and λ_1,*N*_ mean wavelengths of first and *N*th maximum/minimum, respectively.

The measurements were done at temperatures appropriate to BPsI and -II, for a range of wavelengths beyond the Bragg reflection (Figure S9). These experiments are important since theoretical calculations require using these numbers.

**Figure 5:**
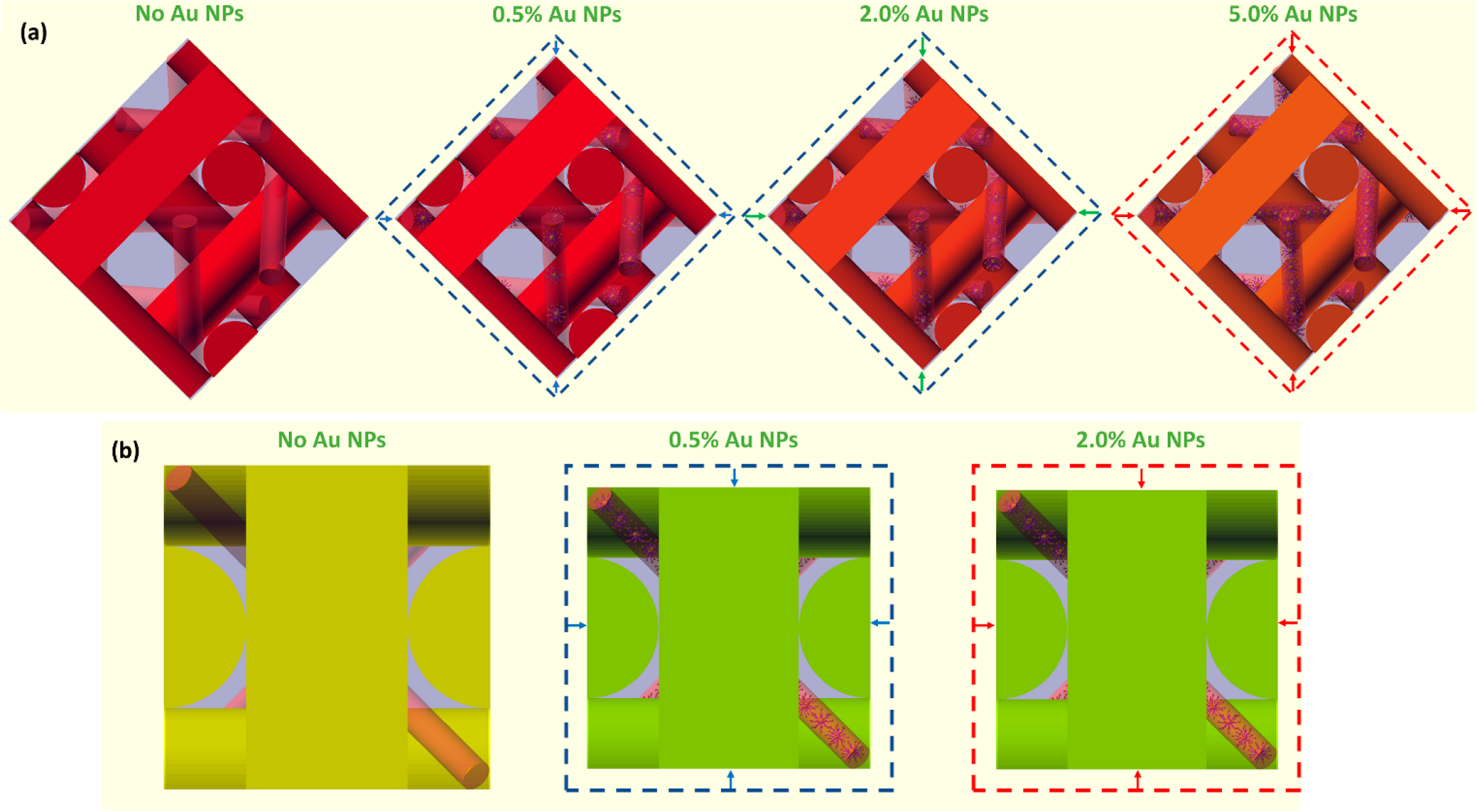
Illustration of the reduction of cubic BP structures in a unit cell by Au NP doping for BPI (110) (a) and BPII (100) (b). The color of DTC structures corresponds to the Bragg wavelength measured in the experiment. Both red dashed lines and arrows correspond to an effect of the saturation threshold reached for which the Bragg reflection changes slightly.

Here, we found that the refractive index of BP increases upon a higher concentration of Au NPs. The change of refractive index of BPI caused by Au doping is on the order of 0.02–0.04 for an NP concentration of 0.5–5.0 wt %. However, the cubic BP lattice structure is reduced by adding Au NPs to the BPLC, which is also illustrated in Figure 5. It turns out that the cubic unit lattice of BPI for undoped BPLC equals 302 nm, while for doped BPLC it decreased as follows: 279 nm (for 0.5 wt %), 263 nm (for 2.0 wt %), and 261 nm (for 5.0 wt %).

Hence, it can be calculated that the relative reductions of the BP unit cell for BPI upon higher NP concentrations of 0.5, 2.0, and 5.0 wt % are as follows: ∼8%, ∼13%, and ∼14%, respectively. The calculations were also performed for BPII. Here, the change of the refractive index in BPII caused by Au doping is less than that for BPI and is of the order of 0.002–0.009 for NP concentrations of 0.5–5.0 wt %. The cubic unit lattice of BPII for undoped BPLC corresponds to 247 nm, while for doped BPLC it slightly decreased as follows: 233 nm (for 0.5 wt %) and 230 nm (for both 2.0 and 5.0 wt %). Thus, the relative reduction of the BP unit cell for BPII upon variation of particle concentration equals ∼6% (for 0.5 wt %) and ∼7% (for both 2.0 and 5.0 wt %). The results of the evaluation of BP lattice constants for all BPLC samples are summarized in Table S1.

**Figure 6:**
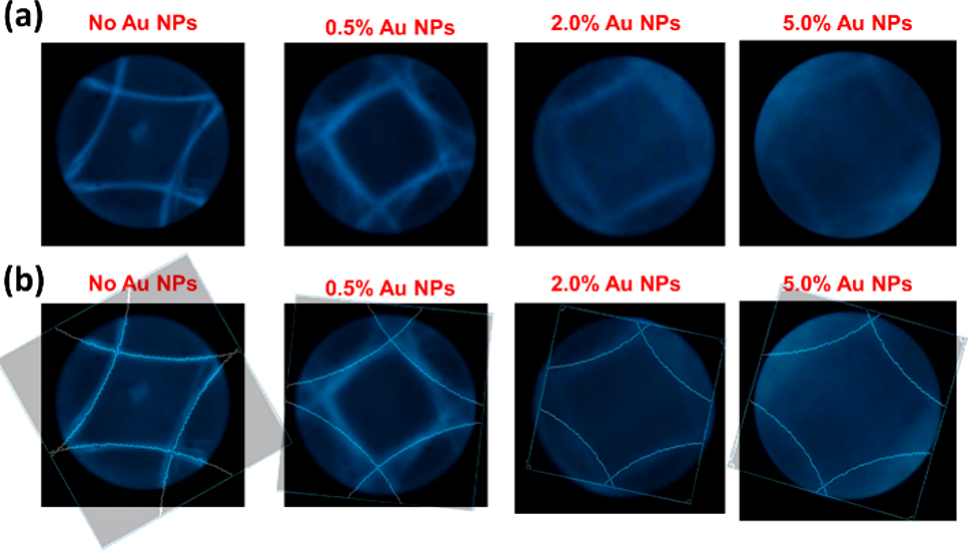
Measurements of the Kossel patterns obtained for BPI (110) doped with Au@L2 NPs for different concentrations (a) and Kossel line fitting (b). The wavelength of 488 nm was used for both the experiment and simulation.

To broaden our analysis of the experimental results, the Kossel diagram of a single unit cell with a lattice orientation of BPI (110) was simulated. The Kossel lines were fitted to the experimental results at the wavelength of 488 nm considering the previously obtained data of the lattice constants (Figure 6). The simulations revealed that, besides a slight variation of the azimuthal crystal plane orientation in BPI, the position of the Kossel lines is further away from the center of the diagram upon variation of particle concentration, still maintaining a (110) crystallographic orientation. Hence, it is proved that a greater reduction of the cubic lattice constant upon a higher concentration of Au NPs occurred (see also Figure S10), simultaneously showing a great agreement between our calculations and the results of the Kossel diagram measurements. This in turn would point out enhancing the chiral twist of DTC structures due to NP doping to the BPLC material.

**Figure 7:**
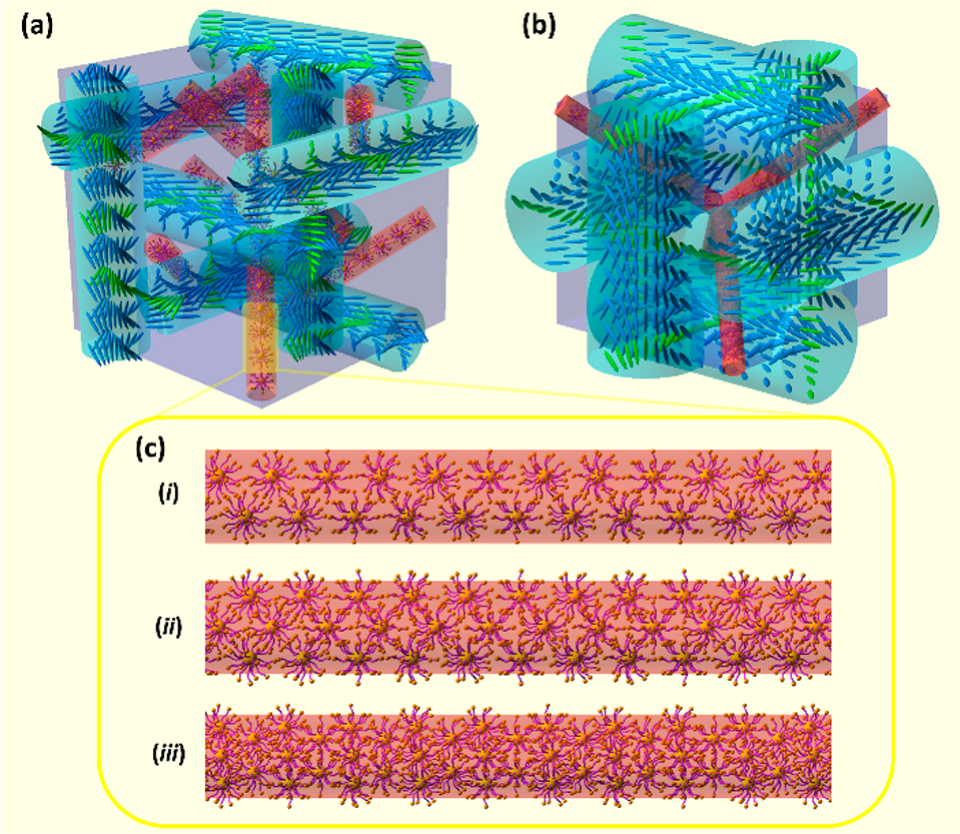
Idealized model of the ordering of LC molecules in the cubic unit cell of BPI (a) and BPII (b) and Au NPs in the LC disclination lines (red rods) for subsequent higher particle concentrations reaching finally a saturation state (c). Green molecules mean connected helices in neighboring cylinders.

We found that the magnitude of a change in relative cell size by NP doping for both BPsI and -II coincides with their cubic lattice constant, which is also illustrated in Figure 7. To understand the difference in NP effect on volume contraction of BPI and BPII, we should recall that the density of packed DTC structures in a cubic unit cell for bcc (BPI) and sc (BPII) equals 0.6802 and 0.5890, respectively.^56^ Specifically, the volume of LC disclination lines in the single unit cell for sc is greater than that for bcc, as can be expressed as follows: *V*_disc_sc_ = 0.4110 and *V*_disc_bcc_ = 0.3198. However, it should be noted that the cubic lattice constant of BPI is 2-fold greater than for BPII because of the periodicity of the helical pitch in a unit cell. Therefore, the ratio of disclination line volume, BPII to BPI, is ∼0.16, showing an evident difference between both BP phases and the feasible influence on their cubic structures by NP doping. This particularly implies that greater coupling between NPs and LC molecules in the bulk occurs for BPI than BPII due to having more space to occupy by NPs in disclination lines.

The detected Bragg wavelength shifting in BPs with NP doping can only be explained by the effect of doping NPs. We know from the TEM measurements that NPs tend to locate at the defect areas and from POM/Kossel that they do not interfere with the formation of the DTC network. The saturated effect of the Bragg wavelength shifting results from the limited space available in the LC disclination lines occupied by small nanoparticles that are different for BPI and BPII (Figure 7a,b). For a relatively small amount of particles, they can accumulate in the LC disclination lines of a BPLC (Figure 7c,i) and interact on the border of disclinations with LC molecules forming a DTC. The LC-like ligands of NPs can penetrate the DTC structures at higher concentrations of Au NPs. Figure 7c,ii, shows a scheme of ligands protruding beyond the area of LC disclinations. Hence, LC molecules forming DTCs would be affected to a larger extent. However, the Bragg wavelength shift is saturated and can reach its maximum for a certain concentration of NP doping, above which only BP stabilization can be achieved due to a dense nanoparticle assembly in the LC disclination core (Figure 7c,iii).

**Figure 8:**
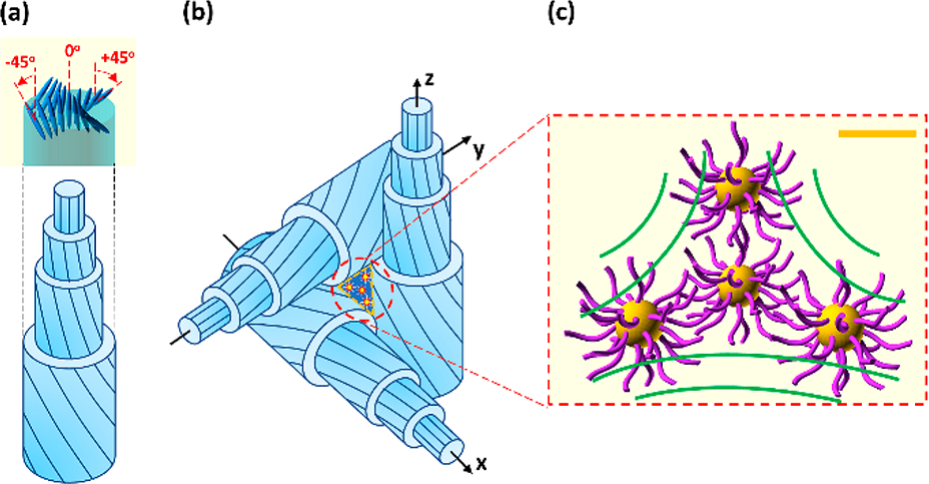
Schematic representation of a DTC unit (a), orientational order of the neighboring DTCs in the volume (b), and the scheme of the possible NP distribution in the area of −1/2 disclination topologically enforced by DTCs (green lines); yellow spheres and violet curves represent Au cores and NP ligands, respectively (c). The blue lines in DTC units indicate the nematic director field. The orange scale bar corresponds to 5 nm.

To briefly discuss how the investigated Au NPs localize in the disclination lines, we need to recall that DTC units (Figure 8a), forming the 3D cubic lattice, cannot provide maintenance of the twisted LC director, topologically creating regions of the −1/2 disclination (Figure 8b). These disclinations have a cylindrical shape with a diameter of the nematic correlation length of ∼10 nm,^39^ which is larger than the size of an NP with toroidal shape of the organic coating (namely, the interlayer distance in NP assemblies without BPLC, ∼7–8 nm). Thus, we expect that more than one NP fits the cross section of the disclination line, and, using scaled dimensions of disclination lines and NPs, we propose a schematic representation of the possible NP distribution in the LC disclination (Figure 8c). It is worth noting that LC-like ligands of NPs can interact with host molecules at the disclination (Supplementary Note 2). Thus, the NP guests reduce the total free energy of the BPLCs by decreasing an energetic cost of the LC disclinations, allowing for packing DTC structures more closely, and finally reducing the lattice constant of the BP unit cell.^39^

Ultimately, we can say that the blue-shifting of the reflection band in doped BPLC results from the interactions of LC-like NP ligands with LC molecules in DTC or the chiral agents; this interaction shortens the DTC helical pitch (in other words, increases twist), reducing the BP unit cell size.

From the application point of view, it is also worth analyzing the effect of NP doping on the thermal properties of the BPLC mixture. In this context, another advantage of doping BPLC with Au@L2 NPs is thermal stabilization of the blue phases, a phenomenon that has been previously described for BPLC-doped materials. Results shown in Figure 4 reveal that the range of temperatures for BPI increased 5.7-fold, from 3.5 °C (54.5–58.0 °C) for undoped BPLC to 19.8 °C (41.0–60.8 °C) for 5.0 wt % of Au NPs in cooling the BPLC samples. For the same sample the temperature range for BPII increased from 2.0 °C (58.0–60.0 °C) to 3.0 °C (60.8–63.8 °C). Additionally, Au@L2 NPs shift the temperature of the BPII–ISO phase transition upon 5.0 wt % of particle concentration (from 60.0 to 63.8 °C), which agrees with a tendency of nematic–isotropic transition temperature shifting observed elsewhere in the composite materials consisting of Au@L2 NPs and LC nematics.^57^ More details on the thermal stabilization of BPLCs by NP doping, considering both heating and cooling the BPLC samples, can be found in Table S1.

Alexander et al. found computationally that the region of stability of the pure cubic BPs depends significantly on the value of the elastic constants.^58^ Notably, the stability of the BP is reduced when the bend elastic constant is larger than splay and when the twist is smaller than the other two. However, the mechanism of extended stabilities of BPs in the investigated samples may be clarified by the reduction of the total free energy of BPLCs by NP guests. The Landau–de Gennes theory can explain this mechanism by considering the free energy density profile *f*(*r*) of BP. The latter can be obtained from the calculated orientational order profile described by a second-rank symmetric and traceless tensor *Q*_*ij*_ at a given temperature and by minimizing the free energy written as follows:^59^
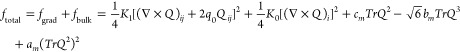
3The first two terms with elastic constants *K*_0_ and *K*_1_ represent the gradient free energy density *f*_grad_ corresponding to the spatial variation of the orientational order. But the last three terms with material parameters *a*_*m*_*, b*_*m*_, and *c*_*m*_ represent the bulk free energy density *f*_bulk_. To check the stability of the BP with a guest component, Fukuda suggested comparing the total free energies of BP and N*, considering both with the same amount of the guest component as follows:^60^
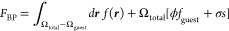
4
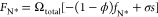
5where Ω_total_ and Ω_guest_ mean the total region and the one replaced by the guest component, respectively; ϕ represents a guest component of the volume fraction; *f*_N*_ is the free energy density of the N* phase; *f*_guest_ is the free energy density of the guest component; σ means the interfacial energy; and *s* means the area of the interface per unit volume.

Then, it can be done by analyzing the free-energy difference per unit volume between BP and N* in the following form:
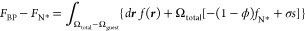
6Equation 6 shows the free-energy difference per unit volume depending on the guest component of volume fraction ϕ and the interfacial energy per unit volume σ. Fukuda calculated that the temperature range of the stability of BPI can become significantly wider by introducing guest components with increasing volume fractions of less than 10%, which coincides with our studies.

The relatively minor increase (only 1.5-fold) of thermal stability of BPII can also be clarified based on a comparison of the different organization of LC disclinations for BPI and BPII, which translates to a saturation effect at lower NP concentration for BPII. Namely, in BPI there are several disclination lines per unit cell that do not intersect, while in BPII all disclination lines connect at the center of the unit cell. Interconnected disclination lines imply that a formed BPII crystal corresponds to a global minimum of free energy. In contrast, in BPI, where the disclination lines are separated, several local minima of free energy exist, since creating or terminating a new disclination line comes at an additional energy cost.^61^ Therefore, the introduction of NPs in the disclination lines should have a stronger impact on BPI than on BPII, because the BPI crystal will reach one of the local minima stabilizing the crystal.

## Conclusions

To conclude, our work shows an efficient way of engineering thermal and optical properties of soft photonic crystals, blue phases, by doping with Au NPs decorated with LC-like ligands. First, we were able to increase the thermal stability of BPs ∼4-fold, from 5.5 °C (for undoped BPLC) up to 22.8 °C (for BPLC-doped at 5.0 wt %). Second, we discovered an unusual effect of shifting the Bragg reflection toward shorter wavelengths in the visible range. Both effects are saturating, and their magnitude depends on the concentration of the investigated nanodopants. The latter effect results from a reduction of 3D BP lattice caused by Au NP doping, with up to ∼14% and ∼7% change of the unit cell size for BPI (110) and BPII (100), respectively. Importantly, the designed surface coating of Au NPs ensures long-term stability of the composite BPs without particle aggregation and without deterioration of large, quasi-monodomain structure formation. The presented results indicate that NP doping BPLC is an efficient and promising method to obtain highly stable BPLCs with adjustable optical properties, making them promising candidates for advanced materials in LC-tunable photonics technology. We believe that our findings will allow us to not only better understand the effect of adjusting photonic properties in multicomponent soft/colloid mixtures with useful optical properties but also improve the fabrication of advanced nanomaterials into 3D periodic soft photonic structures.

## Materials and Methods

### Au NP and Ligand Synthesis

The syntheses of nanoparticles and ligands were conducted following previously described protocols.^45,55^

### Doping NPs into the LC Host

An NP dispersion in toluene was added to a glass vial containing a few milligrams of BPLC, and then, the mixture was thoroughly mixed by sonication at room temperature for 2 min. The volume of the NP dispersion varied for different samples but was always above 10 μL. The required volume of the NP dispersion was calculated using a Au^0^ concentration estimated from sample absorption at 400 nm. After mixing, the vessel was kept open in ambient conditions until toluene evaporated (at least 12 h).

### Preparing BPLC Samples

In the experiment, 12-μm-thick cells were used of high-quality float glass plates with a thickness of 0.7 mm. The glass plates were spin coated with polyimide SE-130 (Nissan Chemical Industries, Ltd.) for homogeneous alignment of LCs. The polyamide-coated substrates were then baked at 80 °C for 30 min and at 180 °C for 1.5 h. Afterward, the substrates were rubbed with a low-pile velvet and calibrated silica spacers of 12 μm were deposited. Finally, flat parallel substrates were assembled as glass cells in antiparallel configuration and sealed with a gasket. Filling glass cells by capillarity with undoped and NP-doped BPLC materials occurred at a high temperature corresponding to the isotropic phase.

### Measurements

LC samples were placed on an mK2000 temperature-controlled stage (Instec) and observed using an Eclipse LV100 POL polarization optical microscope (Nikon) in the reflection mode including a switchable Bertrand lens. To observe the phase sequences of the LC samples, from the isotropic down to the cholesteric phases of LC samples were examined with a cooling rate of 0.2 °C min^–1^. Microscopic images and Kossel diagrams were captured by the DS-Fi1 charge-coupled device (Nikon) that was adapted on POM. Reflection spectra were measured by POM with a USB4000 spectrometer (Ocean Optics). In the Kossel pattern examination, the LC samples placed on a temperature-controlled stage were illuminated by a 488 nm light with a bandwidth of 10 nm. Kossel patterns were observed in the back focal plane of the objective in the microscope by inserting a Bertrand lens. TEM images were acquired using JEOL-1400 (JEOL Co. Japan), equipped with a CCD MORADA G2 high-resolution digital camera (EMSIS GmbH, Germany) available at the Nencki Institute of Experimental Biology of Polish Academy of Sciences, Laboratory of Electron Microscopy. Samples for TEM measurements were prepared by drop-casting of doped BPLC materials on TEM grids coated with carbon film and subsequent heat annealing.

### Kossel Diagram Simulations

Kossel patterns were simulated with Kossel-Kikuchi K-pattern simulation software adjusted for single domains of (110)-oriented body-centered cubic structure with different lattice constants and adapted for a converging monochromatic light of 488 nm. The patterns were simulated and fitted to the experimental results considering the lattice sizes calculated from Bragg wavelengths and the average refractive index of the BPLC samples.

## Supporting Information Available

Experimental details and additional data as noted in the text: temperature dependence of birefringence for the host LC nematic mixture, calculation of the helical pitch for the investigated undoped and NP-doped BPLC samples, size distribution of the investigated Au NPs, chemical structural formula of the investigated LC-like ligands, TEM images of primary Au NPs and NP-doped BPLC samples in N* phase, POM images of undoped and NP-doped BPLC samples, spectra of white-light transmittance through the BPLC samples for different NP concentrations, reflection spectra from the undoped and NP-doped BPLC samples showing central Bragg wavelength for each LC phase and allowing the calculation of their average refractive index, simulations of the Kossel diagrams for (110)-oriented BPI for undoped and NP-doped BPLC samples, and summary of experimental and simulated phase sequences/optical properties of materials used (DOCX)
