# Supplementary material for: Achiral Nanoparticle-Enhanced Chiral Twist and Thermal Stability of Blue Phase Liquid Crystals
Source: ACS Nano. 2022 Dec 8;16(12):20577–88. doi: 10.1021/acsnano.2c07321 (PMC9798865; doi:10.1021/acsnano.2c07321)
Supplement: Supplementary file 1 — nn2c07321_si_001.docx [file nn2c07321_si_001.docx]

Supporting Information

for

**Achiral Nanoparticle-Enhanced Chiral Twist and Thermal Stability of Blue Phase Liquid Crystals**

Kamil Orzechowski,^†*^ Martyna Tupikowska,^‡^ Olga Strzeżysz,^§^ Ting-Mao Feng,^#^ Wei-Yuan Chen,^#^ Liang-Ying Wu,^#^ Chun-Ta Wang,^#^ Eva Otón,^◊^ Michał M. Wójcik,^‡^ Maciej Bagiński,^‡^ Piotr Lesiak,^†^ Wiktor Lewandowski,^‡^ and Tomasz R. Woliński^†^

^†^Faculty of Physics, Warsaw University of Technology, Koszykowa 75, 00-662 Warsaw, Poland

^‡^Faculty of Chemistry, University of Warsaw, Pasteura 1, 02-093 Warsaw, Poland

^§^Institute of Chemistry, Military University of Technology, Kaliskiego 2, 00-908 Warsaw, Poland

^#^Department of Photonics, National Sun Yat-sen University, No. 70 Lien-hai Rd., Kaohsiung 80424, Taiwan

^◊^Institute of Applied Physics, Military University of Technology, Kaliskiego 2, 00-908 Warsaw, Poland

*corresponding author: kamil.orzechowski@pw.edu.pl


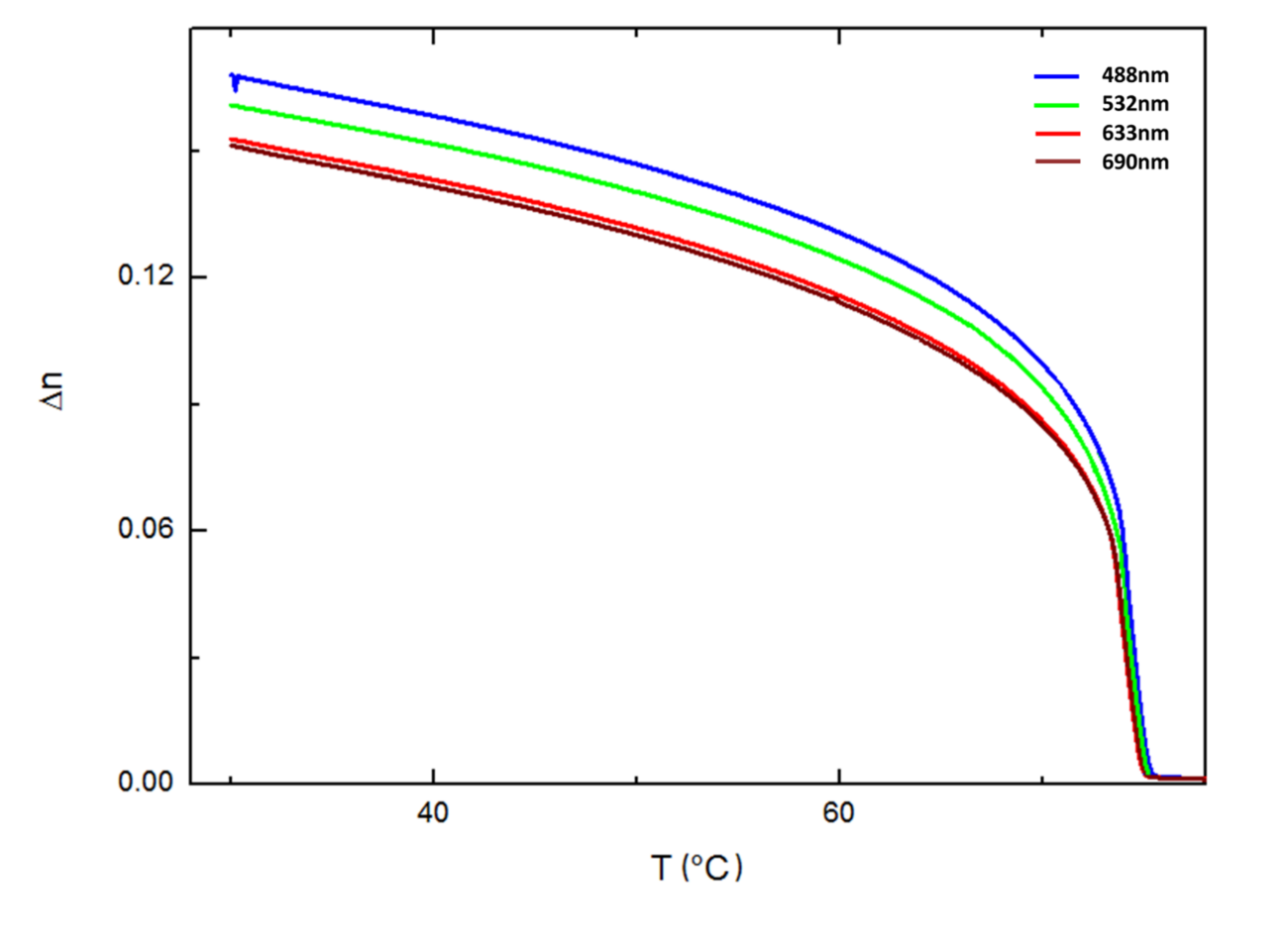


Figure S1. Temperature dependence of birefringence for the host LC nematic mixture measured at four wavelengths from the visible range.


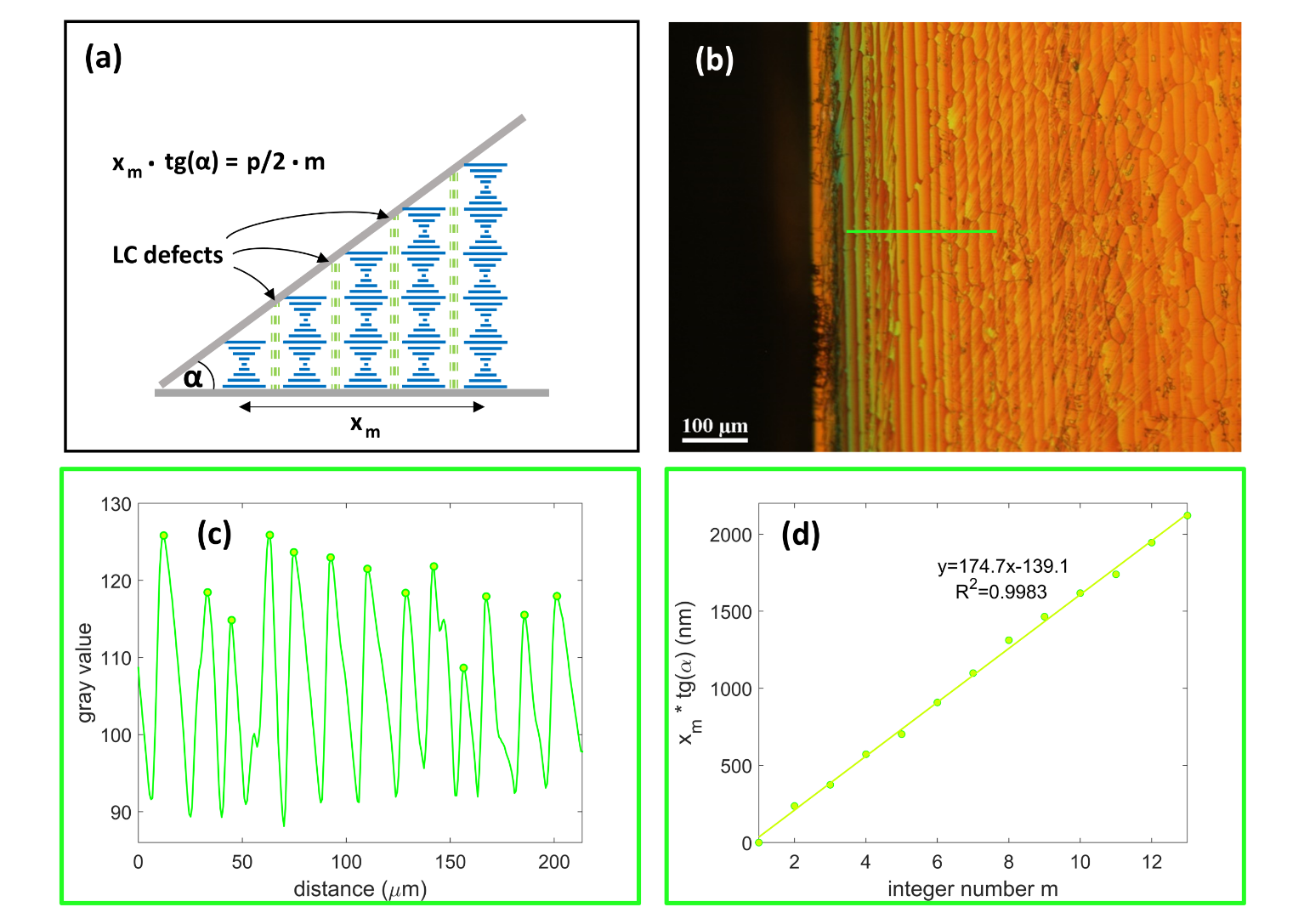


Figure S2. Idealized model of a wedge-cell filled with LC in N* (a), the Grandjean-Cano wedge-cell image of BPLC in N* (b), the characteristic of changing the gray value of pixels along the green line from the image (c), and linear dependence between the subsequent local thickness of LC in a wedge-cell corresponding exactly to an integer number *m* of half-pitches located in the gap (d). The slope of linear dependence means a half pitch of the investigated BPLC sample in N*. The angle of the empty wedge cell was measured optically considering the position of two reflected green laser spots from both plates of the wedge sample and equals 0.64^o^.


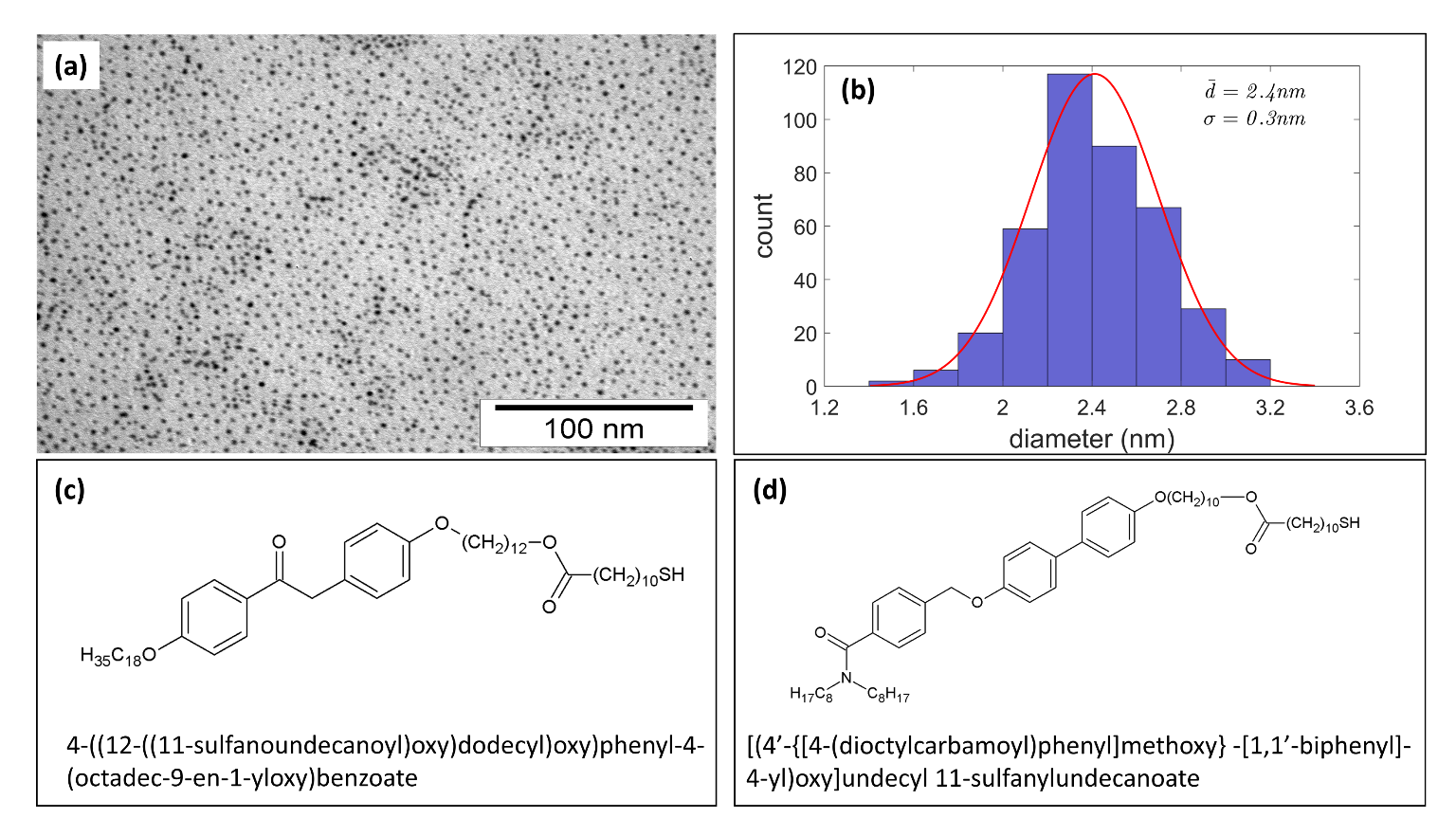


Figure S3. Characterization of the investigated NPs. TEM image of primary Au NPs (a), histogram of the Au NP size distribution (b), and chemical structural formula of the investigated LC-like ligands for L1 (c) and L2 (d), respectively.


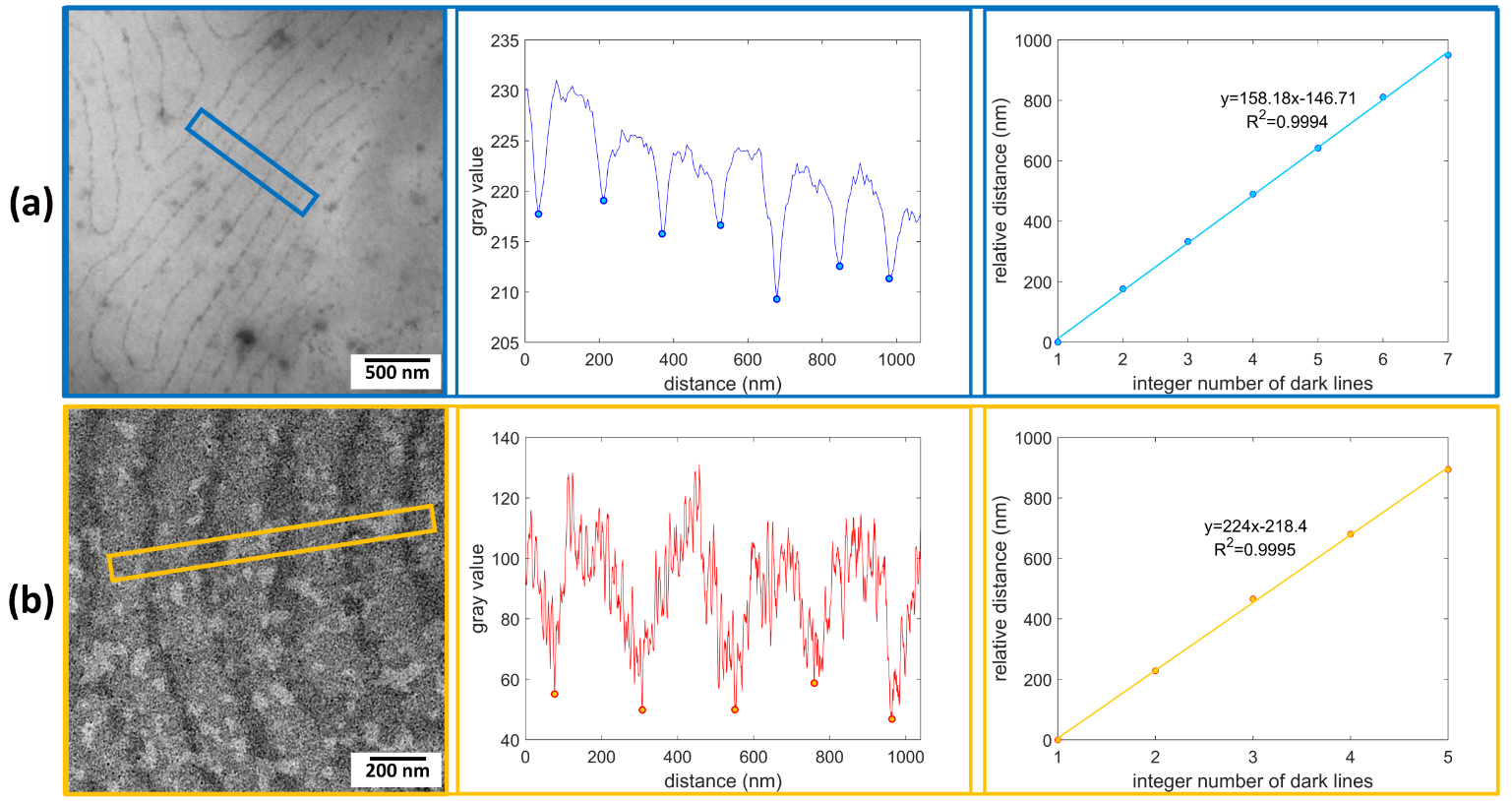


Figure S4. TEM image (in N* phase), the characteristic of changing the gray value of pixels along the length of the rectangle from the image and linear dependence between the subsequent neighboring dark lines for BPLC doped with Au@L1 of 0.5 wt% (a) and Au@L2 of 2.0 wt% (b), respectively. The slope of linear dependence means a half pitch of the investigated NP-doped BPLC samples.


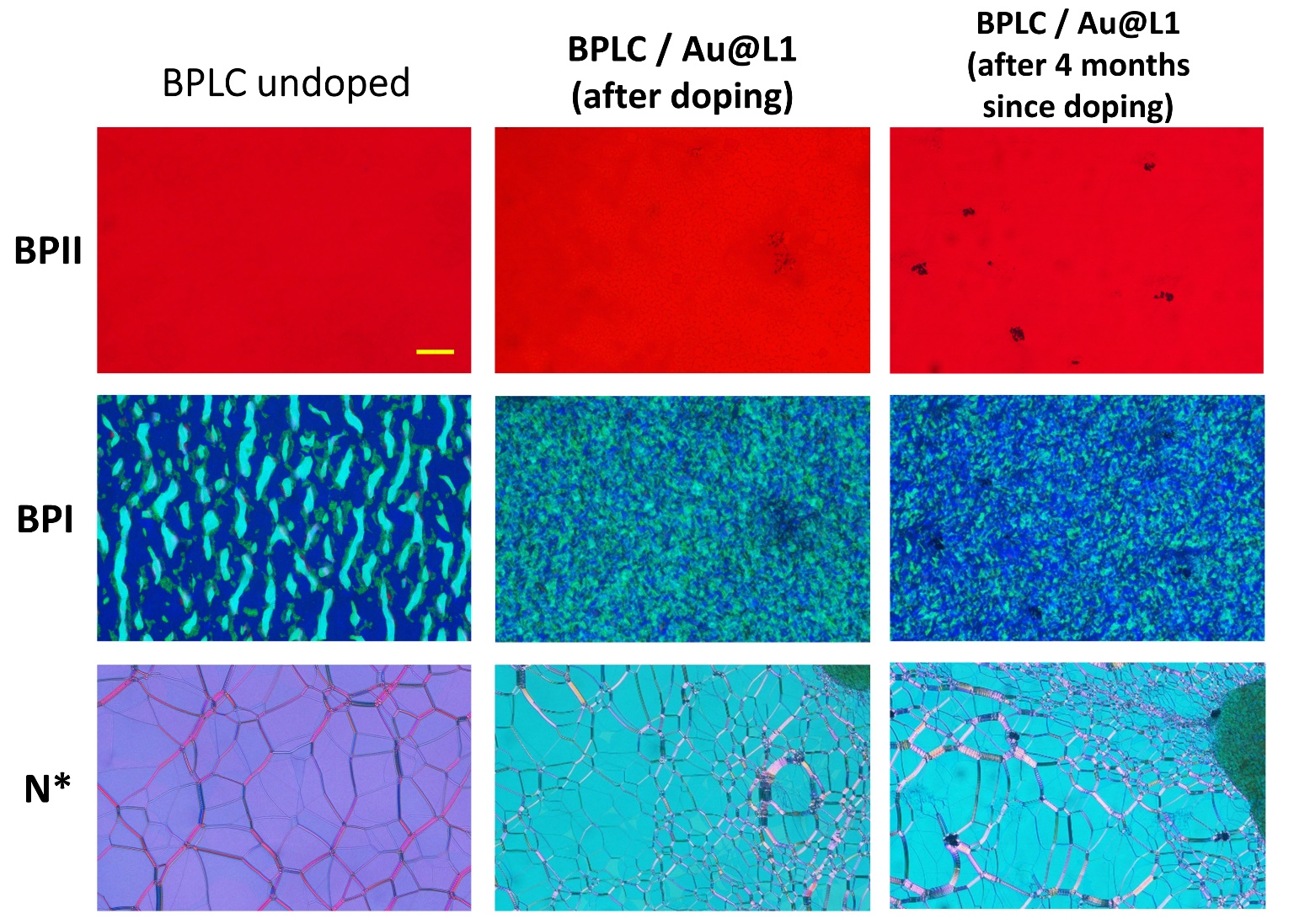


Figure S5. Comparison of BPLC undoped with BPLC doped with Au@L1 NPs. After four months since doping,
a minute NP aggregation occurred locally, visible as small dark spots. The yellow scale bar corresponds to 100 µm.


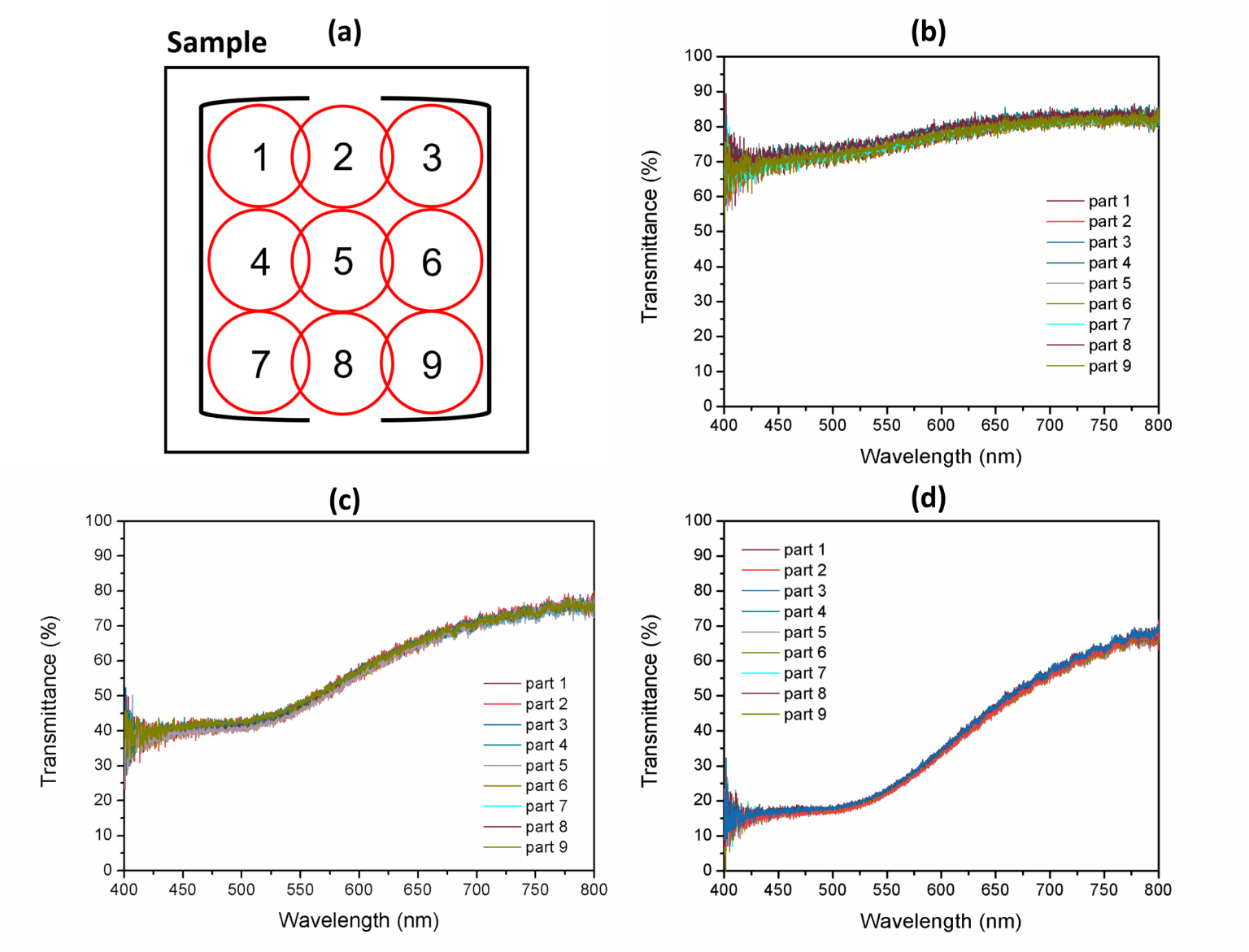


Figure S6. Illustration of the used LC-cell (a), the spectra of white-light transmittance through the Au@L2-doped BPLC samples for different NP concentrations of: 0.5 wt% (b), 2.0 wt% (c) and 5.0 wt% (d). The measurements were done in the isotropic phase and repeated at different parts of the LC-cell to check the uniform of NP dispersibility in BPLC.

Table S1. Summarized results of temperature ranges, Bragg reflection shifting, average refractive index, and lattice unit size obtained at BPI (110) and BPII (100) for undoped BPLC and BPLC doped with Au@L2 of different NP concentrations.


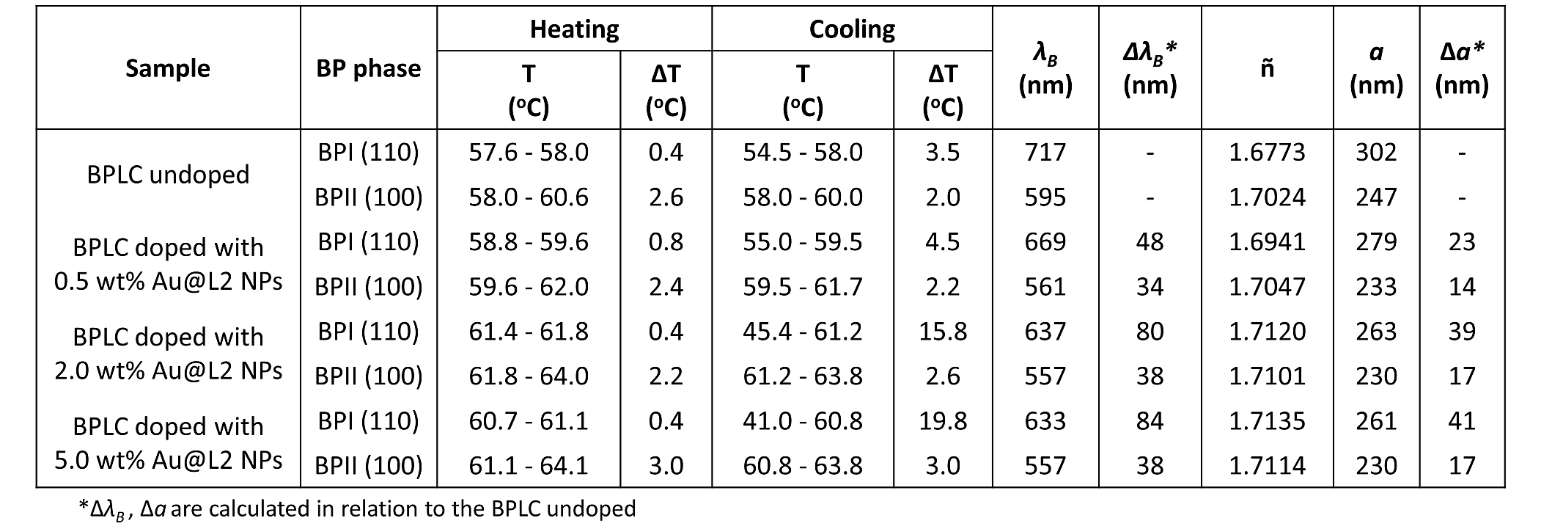


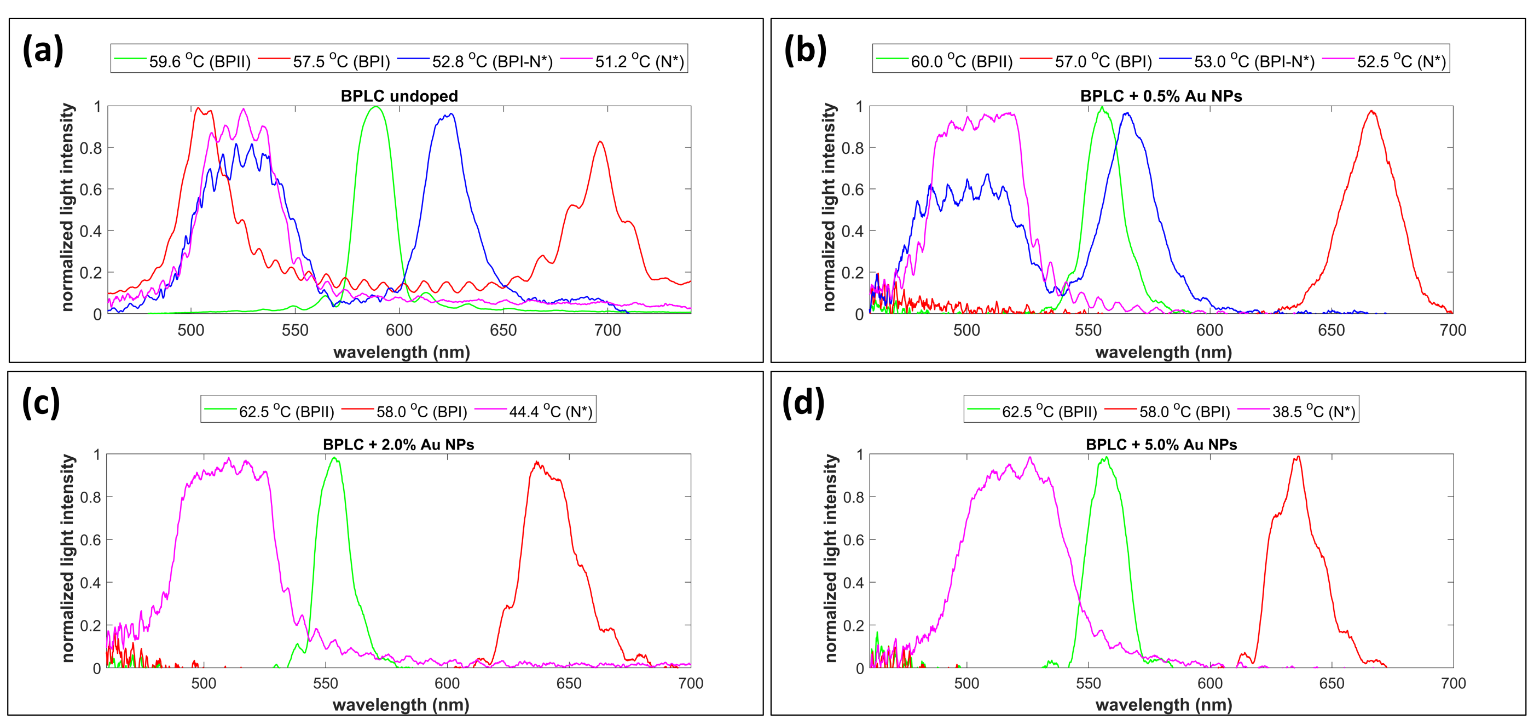


Figure S7. Spectra of white-light reflected from the undoped BPLC (a), and BPLC doped with Au@L2 of the following concentrations: 0.5 wt% (b), 2.0 wt% (c), 5.0 wt% (d). The results show Bragg reflection from the BPLC samples at the selected temperatures appropriate to subsequent LC phases (for the cooling process).

Supplementary note 1:

The experimentally verified saturation threshold of the blue-shift, regarding the tested samples, in N*, BPI, BPII is ~0.5, 2.0, 0.5 wt% of NPs, respectively. However, the magnitude of the blue shift caused by NP doping in N* (from 528 nm up to 503 nm; Δλ_B_ = 25 nm) is smaller than in BPII (from 595 nm up to 561 nm; Δλ_B_ = 34 nm). At the same time the blue shift caused by NP doping at 0.5 wt% in BPI is much greater than for the other LC phases, 48 nm (from 717 nm up to 669 nm). Interestingly, for a higher NP concentration of 2.0 wt%, an obvious red-shift in N* occurs and its value of λ_B_ is also similar at 5.0 wt% of Au NP doped-BPLC. While for BPs, only the blue shift of the Bragg wavelength occurs upon higher Au NP concentration.

Moreover, a similar tendency can be noticed for the helical pitch measurements at N* phase in the investigated BPLC samples. The results were obtained with the use of the Grandjean-Cano method and are presented in Figure S8. They show that the chiral twist is enhanced after adding to the BPLC a relatively low NP concentration of 0.5 wt%. Thus, the helical pitch shortens from 350 nm for the undoped BPLC sample up to 332 nm. However, for a higher NP concentration of 2.0 wt% the pitch is extended from 332 nm up to 345 nm.

These results indicate a clear difference between the NP doping effect occurring in N* compared to BPs, indicating that the strongest coupling between NPs and host molecules is achieved for BPI, while the weakest for N*.


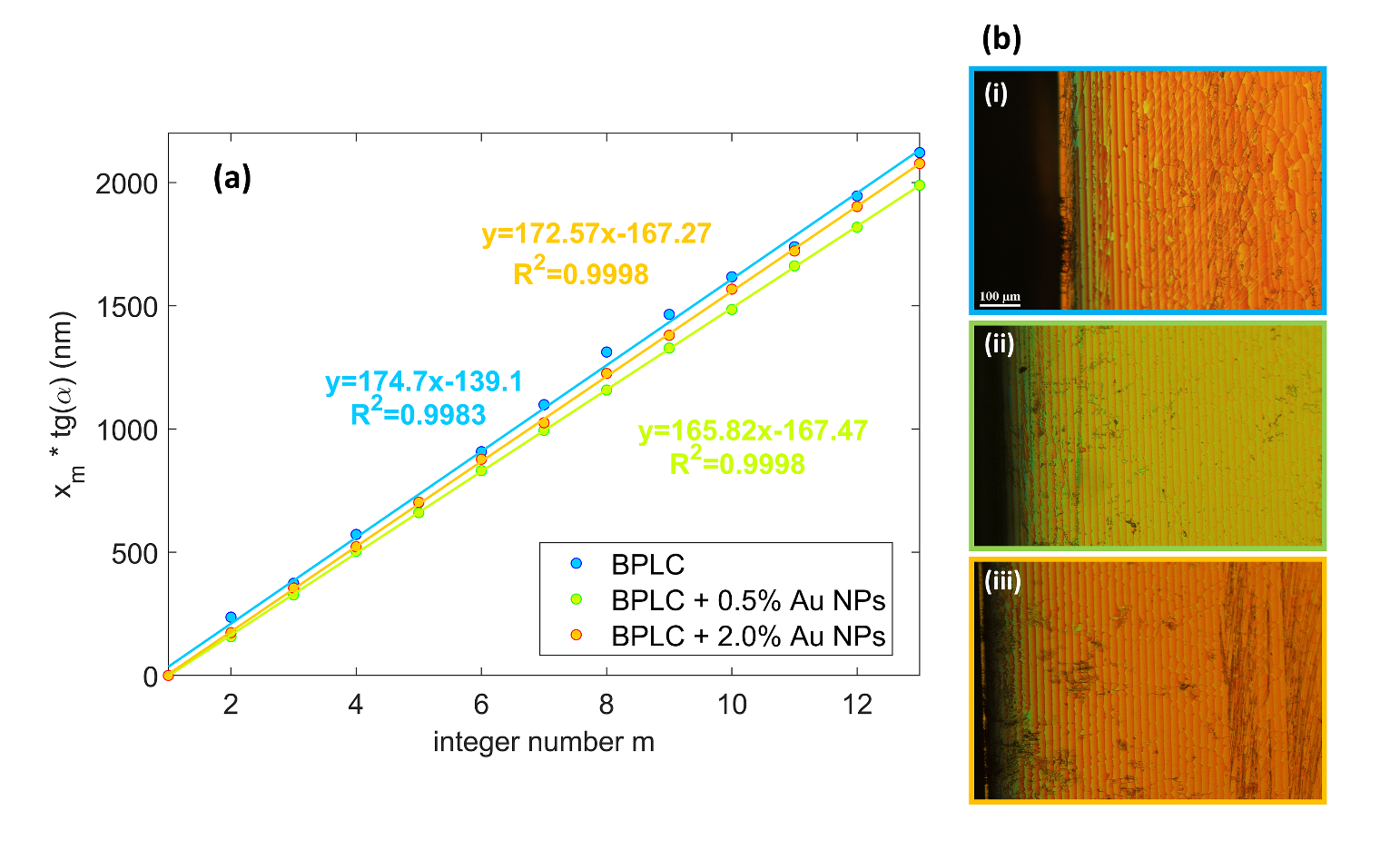


Figure S8. Calculation of the half-pitch (as the slope of linear dependence) for the undoped and NP-doped BPLC samples in N* with the use of the Grandjean-Cano wedge-cell method (a), and POM images of BPLC undoped (i), and doped-BPLC samples for NP concentration of 0.5 wt% (ii) and 2.0 wt% (iii) recorded in reflection (b). The angles of the empty wedge cells were measured optically considering the position of two reflected green laser spots from both plates of the wedge samples and equal 0.64^o^ for undoped BPLC and 0.31^o^ for both NP-doped BPLC samples.


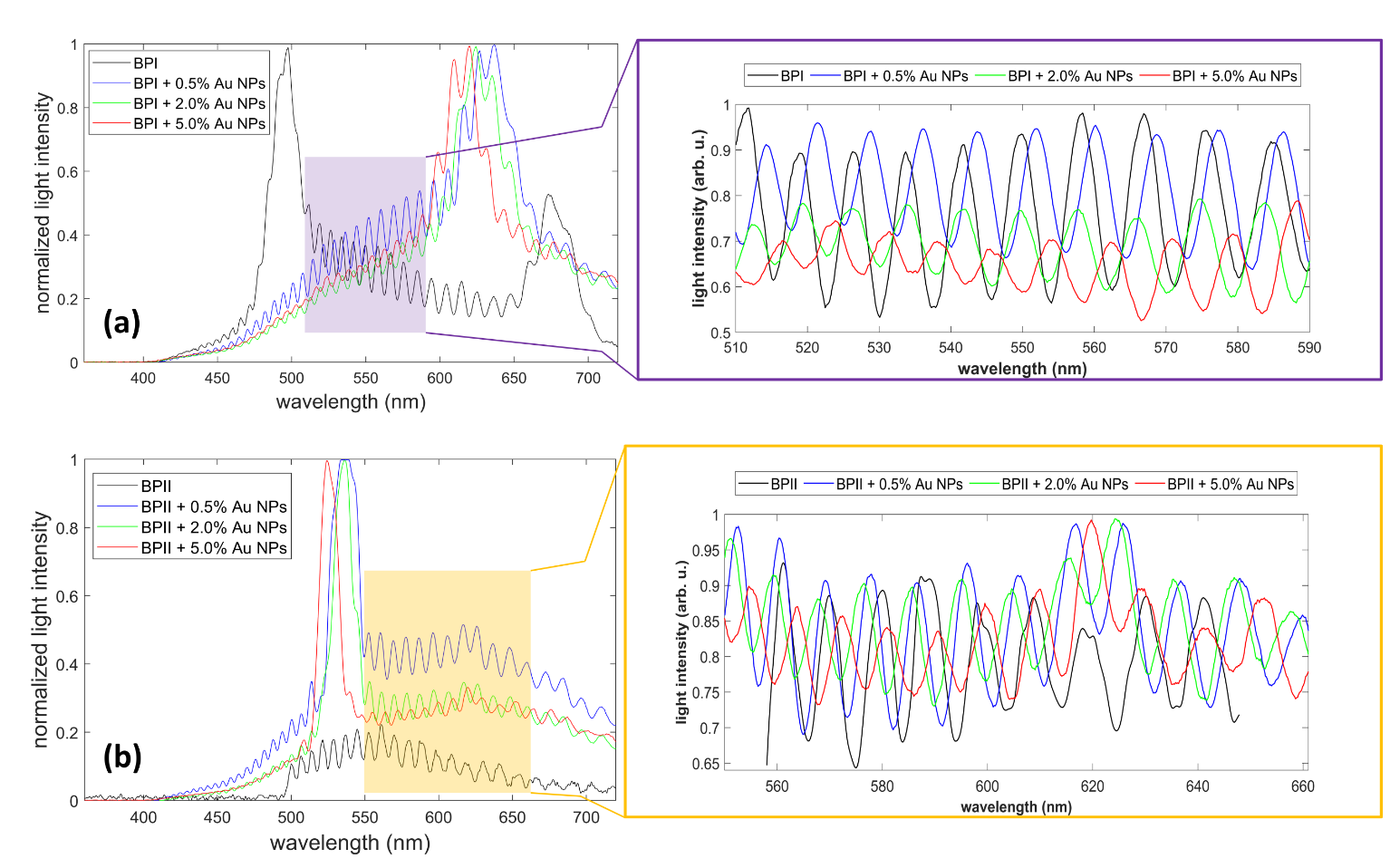


Figure S9. Spectra of white light reflected from the undoped and Au NPs-doped BPLC samples for calculating their average refractive index based on modulated signal covering the range of wavelengths beyond the Bragg reflections at BPI (a) and BPII (b). The measurement for the undoped BPII was taken close to BPII-ISO phase transition before a clear peak of Bragg reflection occurred. The characteristics from the selected range of wavelengths were flattened and grouped to ensure a fair comparison of the results.


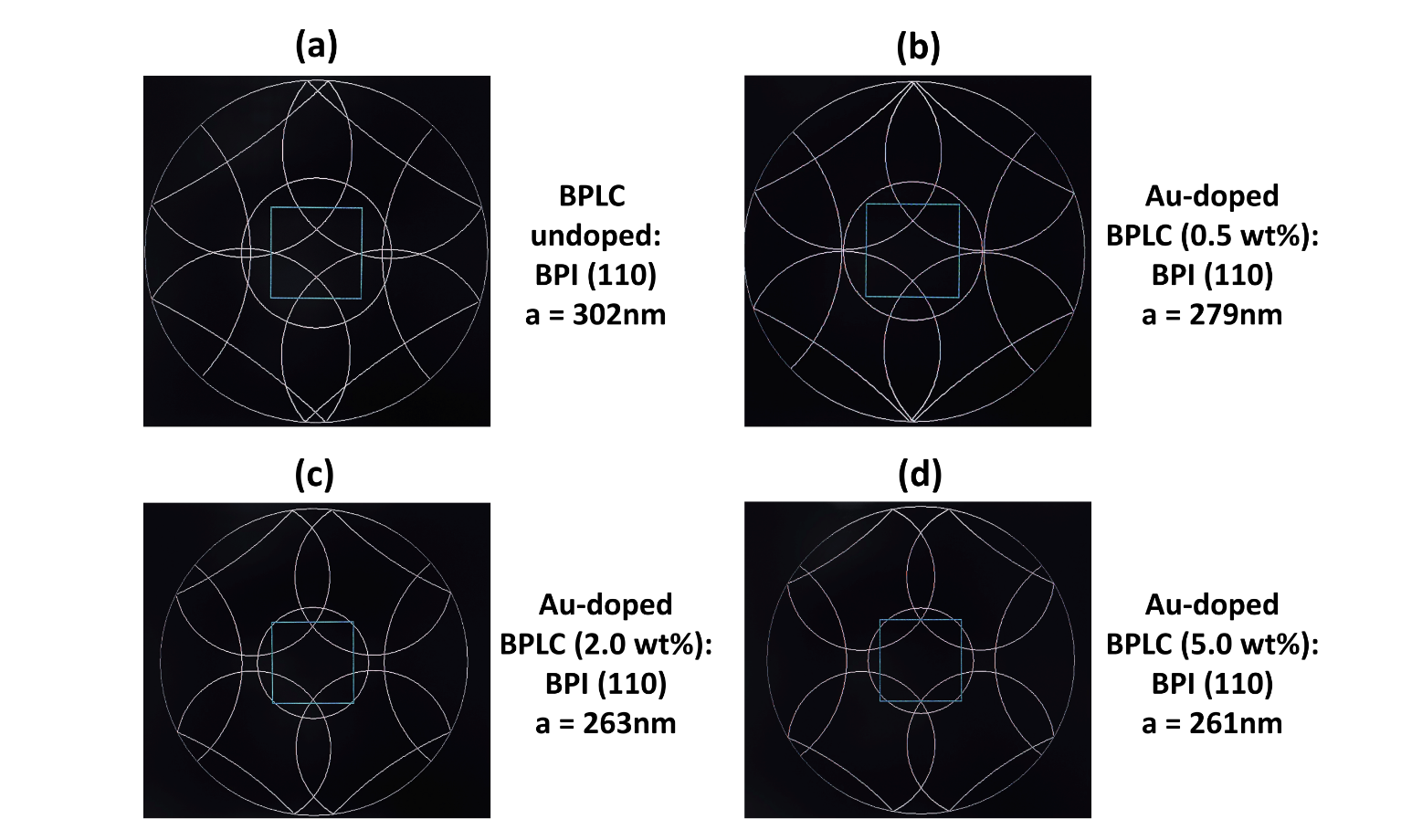


Figure S10. Simulations of the Kossel diagrams for BPI (110) for undoped (a) and Au doped-BPLC samples for different Au NP concentrations of: 0.5 wt% (b), 2.0 wt% (c) and 5.0 wt% (d). The cyan square means the area of observations under a microscope in the experiment. Simulations were done at wavelength of 488 nm.

Supplementary note 2:

Regarding nanoparticle interactions with BP matrix molecules, we can say that the used binary monolayer of thiols (dodecanethiol and liquid crystal-like ligand) was previously shown to enable an efficient formation of assemblies of metallic nanoparticles in liquid crystalline hosts. In such systems nanoparticles usually occupy LC defects, similar to the case described here. To fully understand how organic shell composition translates to the efficient formation of NPs/LCs composites, two major matters should be discussed. On one side, binary composition of the ligand shell ensures an efficient mixing of nanoparticles with the matrix in the isotropic phase (at an elevated temperature): (a) chemical structure of liquid-crystal like ligand ensures π-π and dipol-dipol interactions with the host matrix molecules, (b) rotational freedom of longer ligands enhances colloidal stability of nanoparticles (Nat. Commun. 2019, 10, 2454; 10.1002/adfm.201805094). On the other side, the selective placement of nanoparticles at the defect lines (disclinations) can be understood in the view of: (a) the presence of alkyl (shorter) ligands limits the steric hindrance within the organic shell of nanoparticle, increasing its flexibility and leading to tactoidal deformation (anisotropic shape of the organic shell around metallic spheres), thus, we can hypothesize that the organic shell is able to deform to adapt to the geometry of the disclination line, (b) alkyl ligands can act as a spacer creating supramolecular pockets for the penetration of matrix molecules, further enhancing interactions with the host molecules, (c) placing of nanoparticles in defects should limit the energy fault due to admixing of nanoparticle ligands with the host molecules. These considerations are crucial for the success of interfacing structured matrices, such as liquid crystalline systems, with nanoparticles.
